# Extracellular and Intracellular Angiotensin II Regulate the Automaticity of Developing Cardiomyocytes *via* Different Signaling Pathways

**DOI:** 10.3389/fmolb.2021.699827

**Published:** 2021-08-25

**Authors:** Zenghua Qi, Tao Wang, Xiangmao Chen, Chun Kit Wong, Qianqian Ding, Heinrich Sauer, Zhi-Feng Chen, Cheng Long, Xiaoqiang Yao, Zongwei Cai, Suk Ying Tsang

**Affiliations:** ^1^ School of Life Sciences, The Chinese University of Hong Kong, Shatin, Hong Kong, SAR China; ^2^ Institute of Environmental Health and Pollution Control, School of Environmental Science and Engineering, Guangdong University of Technology, Guangzhou, China; ^3^ State Key Laboratory of Environmental and Biological Analysis, Department of Chemistry, Hong Kong Baptist University, Kowloon, Hong Kong, SAR China; ^4^ School of Life Sciences, South China Normal University, Guangzhou, China; ^5^ Department of Physiology, Justus Liebig University Giessen, Giessen, Germany; ^6^ School of Biomedical Sciences, The Chinese University of Hong Kong, Shatin, Hong Kong, SAR China; ^7^ Key Laboratory for Regenerative Medicine, Ministry of Education, The Chinese University of Hong Kong, Shatin, Hong Kong, SAR China; ^8^ State Key Laboratory of Agrobiotechnology, The Chinese University of Hong Kong, Shatin, Hong Kong, SAR China

**Keywords:** developing cardiomyocytes, angiotensin II, angiotensin II receptor, spontaneous action potential, calcium

## Abstract

Angiotensin II (Ang II) plays an important role in regulating various physiological processes. However, little is known about the existence of intracellular Ang II (iAng II), whether iAng II would regulate the automaticity of early differentiating cardiomyocytes, and the underlying mechanism involved. Here, iAng II was detected by immunocytochemistry and ultra-high performance liquid chromatography combined with electrospray ionization triple quadrupole tandem mass spectrometry in mouse embryonic stem cell–derived cardiomyocytes (mESC-CMs) and neonatal rat ventricular myocytes. Expression of AT_1_R-YFP in mESC-CMs revealed that Ang II type 1 receptors were located on the surface membrane, while immunostaining of Ang II type 2 receptors (AT_2_R) revealed that AT_2_R were predominately located on the nucleus and the sarcoplasmic reticulum. While extracellular Ang II increased spontaneous action potentials (APs), dual patch clamping revealed that intracellular delivery of Ang II or AT_2_R activator C21 decreased spontaneous APs. Interestingly, iAng II was found to decrease the caffeine-induced increase in spontaneous APs and caffeine-induced calcium release, suggesting that iAng II decreased spontaneous APs *via* the AT_2_R- and ryanodine receptor–mediated pathways. This is the first study that provides evidence of the presence and function of iAng II in regulating the automaticity behavior of ESC-CMs and may therefore shed light on the role of iAng II in fate determination.

## Introduction

The renin–angiotensin system (RAS) plays a central role in the regulation of water balance and blood pressure; in addition, it also has an important role in the cardiovascular system (Harada et al., 1999). Angiotensin II (Ang II) is the key bioactive molecule of RAS; it is produced either systemically or locally *via* the proteolytic processing of angiotensinogen to angiotensin I (Ang I) by renin, followed by the subsequent conversion of Ang I to Ang II by angiotensin converting enzyme (ACE) or chymase (Danser et al., 1999; Paul et al., 2006). Ang II, the major effector of RAS, was found to directly affect the contractility and the metabolism of adult cardiomyocytes (CMs) and is responsible for hypertrophy (Baker et al., 1992). Two distinct Ang II receptor subtypes, namely, the Ang II type 1 receptor (AT_1_R) and the Ang II type 2 receptor (AT_2_R), have been identified. Both AT_1_R and AT_2_R belong to the G protein–coupled receptor (GPCR) superfamily, but they are found to induce different cellular signaling pathways (Akazawa et al., 2013; Karnik et al., 2015). Numerous studies have demonstrated that AT_1_R activation can lead to disease states including hypertension, cardiac arrhythmia, stroke, diabetic nephropathy, and metabolic disorders (de Gasparo et al., 2000; Zaman et al., 2002; Thomas and Mendelsohn, 2003). AT_1_R-null mice appear to show a lower risk of cardiovascular disease (Harada et al., 1999). The most classical pathway of AT_1_R is dependent on heterotrimeric G proteins. AT_1_R-Gq_/11_-phospholipase Cβ (PLCβ) coupling leads to the production of inositol trisphosphate (IP_3_) and diacylglycerol (DAG) (Yusuf et al., 2000), which can activate the inositol 1,4,5-trisphosphate receptor (IP_3_R) and the transient receptor potential (TRP) canonical 3 and 6 (TRPC3 and TRPC6) channels, respectively, to increase the concentration of cytosolic Ca^2+^ ([Ca^2+^]_i_) (Onohara et al., 2006). Although AT_2_R shares ∼34% amino acid sequence homology with AT_1_R, AT_2_R is markedly different from AT_1_R in terms of tissue-specific expression, signaling mechanisms, receptor function regulation, and pharmacological properties (Jones et al., 2008; Karnik et al., 2015). According to previous studies, AT_2_R can attenuate the detrimental effects of AT_1_R to protect the heart from disease development (Padia and Carey, 2013; Inuzuka et al., 2016). AT_2_R has been proven to be a GPCR, with all the classical motifs and signature residues of a GPCR (Lokuta et al., 1994; Gallinat et al., 1999; Asada et al., 2018). However, it is still unclear whether AT_2_R can activate a classical G protein signaling pathway.

AT_1_R and AT_2_R were traditionally thought to be presented on the plasma membrane (George et al., 2010; Savoia et al., 2011). However, Ang II was also shown to bind to the nuclear membrane and nuclei of CMs (Baker et al., 1992), suggesting the existence of intracellular Ang II (iAng II) receptors. In addition, previous studies have shown that AT_2_R was strongly detected in the perinuclear region or in the nuclei of CMs (Senbonmatsu et al., 2003; Tadevosyan et al., 2010), hinting that iAng II may act in an intracrine manner through AT_2_R. Although there were previous studies showing that exogenous application of Ang II increases the contraction amplitude and frequency of CMs (Sedan et al., 2008; Lagerqvist et al., 2011), it is unclear whether iAng II exerts any effect in early developing CMs, which, unlike adult CMs, uniquely display automaticity, and the signaling pathways involved.

Automaticity is a fundamental physiological feature of the pacemaker cells in the heart; it is characterized by the existence of spontaneous phase 4 diastolic depolarization (DD) in an action potential (AP). It is now recognized by numerous researchers in the pacemaker field that the two clocks (the membrane clock and the calcium clock) function in a coupled system synergistically to ensure the automaticity (Lakatta et al., 2010; Monfredi et al., 2013). The membrane clock is attributed by an interplay of a number of ion channels, pumps, and exchangers, such as the L-type Ca^2+^ channel, T-type Ca^2+^ channel, delayed rectifier K^+^ channel, hyperpolarization-activated cyclic nucleotide-gated channels, sodium–calcium exchanger, and Na^+^/K^+^-ATPase. Besides the membrane clock, spontaneous calcium release from the sarcoplasmic reticulum (SR) is another determinant of automaticity. Local calcium releases (LCRs) are the elementary SR calcium releases mediated by the calcium release unit (CRU), which is composed mainly of ryanodine receptor isoform 2 (RyR2) but also IP_3_ receptor (IP_3_R) (Cheng and Lederer, 2008). LCRs can occur spontaneously in CMs independent of the calcium influx through the L-type Ca^2+^ channel, as LCRs continue even when the L-type Ca^2+^ channel is blocked pharmacologically (Cheng and Lederer, 2008). Calcium release from the SR and calcium refill into the SR are regarded as the calcium clock.

Apart from its role in maintaining the LCR, another important role of RyR2 is its contribution to Ca^2+^-induced Ca^2+^ release (CICR). Upon the arrival of AP, plasma membrane depolarization opens L-type Ca^2+^ channels; the calcium influx *via* L-type Ca^2+^ channels would in turn trigger the opening of RyR2, leading to CICR. This calcium increase is the link for excitation–contraction coupling in CMs.

While it has been known for a long time that calcium is the activator of RyR2 and that ryanodine is a widely used pharmacological blocker of RyR2, the physiological inhibitory pathway of RyR2 has not been clearly identified. Whether there would be any upstream signaling pathway which would eventually lead to the inhibition of RyR2, either directly or indirectly, is unexplored.

While some recent studies revealed the function of iAng II in the cardiovascular system (De Mello, 2015; Tadevosyan et al., 2015; Tadevosyan et al., 2017), it remains unclear whether iAng II could modulate the automaticity of developing CMs [embryonic stem cell–derived CMs (ESC-CMs) and neonatal rat ventricular myocytes (NRVMs)]. All in all, the existence and the function of iAng II and AT_2_R in automatically firing CMs are unknown. Because of the pivotal role of RyR2 in these CMs, it would be important to determine if iAng II, upon the activation of AT_2_R, if any, would act *via* RyR2 to exert its effect. The aims of this study were 1) to investigate if iAng II and its receptors are present in developing CMs, 2) to investigate if extracellular and iAng II regulate the spontaneous APs of developing CMs and exert their effects differentially, and 3) to elucidate the signaling pathways through which iAng II regulates spontaneous APs of developing CMs.

## Materials and Methods

### Mouse Embryonic Stem Cell Culture and Differentiation of Mouse Embryonic Stem Cells

Culture of mESC cell line D3 (ATCC, Manassas, VA, United States, 28 passages) and differentiation of mESCs using the hanging drop method were performed as we have previously described (Ng et al., 2010; Wong et al., 2012; Law et al., 2013; Qi et al., 2016). A full description of the methods is available in “Supplementary Materials and Methods.”

### Isolation of Neonatal Rat Ventricular Cardiomyocytes

NRVMs were isolated from neonatal Sprague-Dawley rats (1- to 2-day-old) using a previously described method (Wollert et al., 1996). Briefly, the rat hearts were dissected out and digested with trypsin. The dissociated cells were then suspended in DMEM/F12 with GlutaMAX (Gibco, Grand Island, NY, United States) supplemented with 10% horse serum (Gibco), 5% FBS (Gibco), and 50 μg/ml gentamicin (Gibco). The isolated cells, which were still heterogeneous, were then plated to cultureware in an incubator for 60 min to allow the removal of cardiac fibroblasts as they would selectively adhere to the cultureware. Nonadhesive NRVMs which were still in suspension were transferred and plated on glass coverslips pre-coated with Matrigel (Corning, Corning, NY, United States) for further culture.

Quantification of iAng II levels in NRVMs by ultra-high performance liquid chromatography combined with electrospray ionization triple quadrupole tandem mass spectrometry (UHPLC-ESI-MS/MS) was carried out.

NRVMs (2 × 10^6^ cells) from 10 neonatal rats were lysed in ice-cold RIPA buffer freshly supplemented with a protease inhibitor cocktail and placed on ice for 15 min and centrifuged at 16,000 *g* at 4°C for 20 min. The supernatant (300 μl) that contained the protein was stored at −80°C as the next procedure sample. Protein concentration was determined using Bradford assay with bovine serum albumin (BSA) as the standard. For the analysis of iAng II, a sample (300 μl) was subsequently extracted using C18 Sep-Pak Vac 3cc cartridges (200 mg) (Waters, Milford, MA, United States), purified by immunoaffinity chromatography with the immobilization of the anti–Ang II antibody on CNBr-activated Sepharose 4B cartridges (GE Healthcare, Uppsala, Sweden), and analyzed using an Ultimate 3000 UHPLC system coupled with a Thermo TSQ mass spectrometer (Thermo Fisher Scientific, Waltham, MA, United States). The double-charged parent ions of the native Ang II were *m*/*z* 523.8 ± 0.1 (Supplementary Figure S1A). The structure of Ang II was confirmed by two kinds of MRM transitions, which were *m*/*z* 523.8 ± 0.1 → *m*/*z* 263.1 ± 0.1 and *m*/*z* 523.8 ± 0.1 → *m*/*z* 784.4 ± 0.1 (Supplementary Figures S1B,C). The former fragment ion was the dominant ion with the highest intensity, so that it was chosen as the quantitative ion, while the latter one was used as the qualitative ion. The coefficient of determination r^2^ for validation was found to be 0.9993. The detection limit and the lower limit of quantification were determined to be 0.1 and 0.3 ng/ml, respectively. The detailed procedures for the quantification of iAng II levels in NRVMs by UHPLC-ESI-MS/MS are described in “Supplementary Materials and Methods.”

### Electrophysiology

For single-cell patch clamp recording, glass coverslips containing single cells were placed onto a recording chamber with temperature control (33°C) and perfused with Tyrode’s solution. The pipette solution referred to the components in the previous articles (Muller et al., 1997; Qi et al., 2016). Action potentials were recorded using an Axopatch 200B amplifier (Molecular Devices, Sunnyvale, CA, United States) and pCLAMP 10.4 software (Molecular Devices), using rupture whole-cell patch-clamp in current-clamp configurations.

To investigate the effect of iAng II on the automaticity of CMs, we developed a dual patch clamp method which was performed as follows: one electrode was designated as the recording electrode, and the other electrode was designated as the drug delivery electrode. GΩ seals were acquired with both electrodes on the same cell. The cell membrane patched by the recording electrode was first broken without breaking the membrane patched by the drug delivery electrode. Baseline APs of CMs were recorded for 3 min. Thereafter, the cell membrane patched by the drug electrode was ruptured to allow drug delivery into cells and APs of CMs were simultaneously recorded by the recording electrode. The bath solution and pipette solution of the recording electrode were the same as those in single patch clamp. Pipette solution of the drug delivery electrode was composed of pipette solution and drugs. Clampfit 10.4 (Molecular Devices) and GraphPad Prism 5 (GraphPad Prism Software, La Jolla, CA, United States) were used to analyze the recorded data.

### Measurement of [Ca^2+^]_i_

For detecting the effect of iAng II on the [Ca^2+^]_i_, 5 µM Fluo-4 (Invitrogen) was loaded into the single mESC-CMs. Thereafter, whole-cell patch-clamp was performed with pipette solution containing Ang II and bath solution that was Tyrode’s solution. After the establishment of the current-clamp mode, the bath solution was gently changed to Ca^2+^-free solution. Subsequently, cells were imaged using a CCD camera (Photometrics, Tucson, AZ, United States). Images were acquired at a frequency of 35 Hz using a MetaFluor/MetaMorph Imaging System (Molecular Devices).

### Statistical Analysis

Each experiment was repeated at least three times, and the data were shown as mean ± SEM. Unpaired Student’s *t*-test and analysis of variance (ANOVA) were applied to take the statistical significance between two groups and three groups or more, respectively. *p* < 0.05 was considered to be statistically significant.

## Results

### iAng II Was Detected in Developing Cardiomyocytes

**FIGURE 1:**
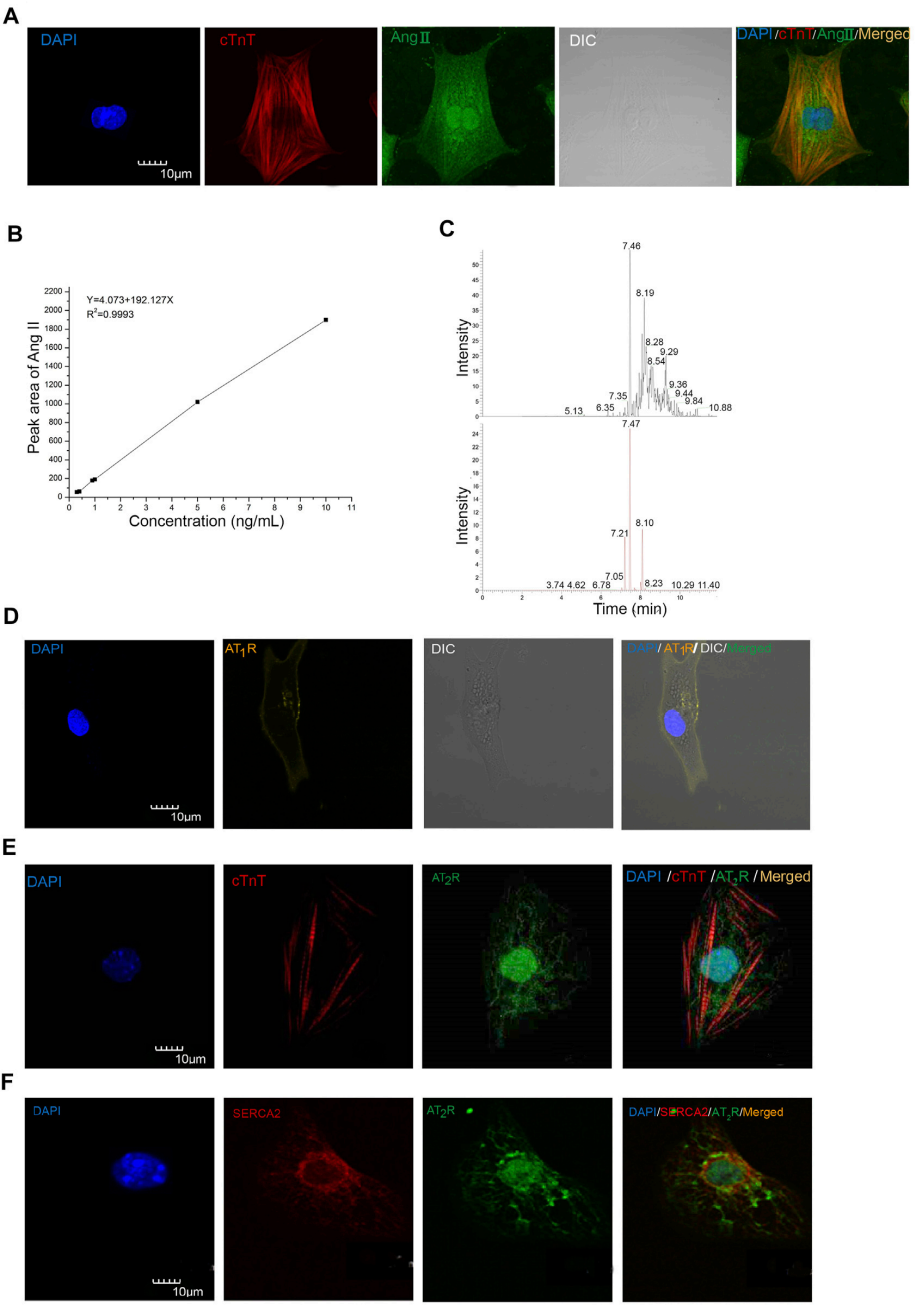
Ang II, AT_1_R, and AT_2_R were detected in mESC-CMs and NRVMs. **(A)** Representative images showing the immunocytochemistry of iAng II in mESC-CMs. Cells were immunostained for cTnT (red), Ang II (green), and the nucleus with DAPI (blue). The results indicated that Ang II was present in mESC-CMs. **(B)** Linear calibration curve of Ang II. **(C)** Analysis of the NRVM samples by UHPLC-ESI-MS/MS. The extracted ion chromatograms of the two kinds of MRM. The upper one is the 523.8 ± 0.1 m/z →263.1 ± 0.1 m/z; the lower one is the 523.8 ± 0.1 m/z →784.4 ± 0.1 m/z. **(D)** mESC-CMs were transduced with the vector carrying AT_1_R-YFP and were stained with DAPI (blue). AT_1_R-YFP signals were detected on the plasma membrane. **(E, F)** mESC-CMs were stained with DAPI (blue), anti-AT_2_R (green), and **(E)** anti-cTnT (red) or **(F)** anti-SERCA2 (red). Merged images revealed extensive co-localization of AT_2_R with the nucleus and the SERCA2, suggesting that AT_2_R was located predominately on the nucleus and the SR. Scale bars represent 10 μm.

The iAng II was detected by immunocytochemistry and our newly developed method by UHPLC-ESI-MS/MS in mESC-CMs and NRVMs, respectively. First, we examined whether Ang II is present in mESC-CMs by immunostaining, followed by confocal fluorescence microscopy. Positive staining with cardiac tropinin T (cTnT), a CM-specific marker, confirmed that the cells were CMs. Ang II was detected in the cytoplasm of mESC-CMs (Figure 1A). Furthermore, the result of UHPLC-ESI-MS/MS analysis revealed that Ang II could be detected in NRVMs, and the concentration of Ang II in the NRVM sample was 0.79 ng/ml (Figures 1B,C). The linearity range of the native Ang II was determined. The six-point calibration curve of Ang II showed a reliable reproducibility in the concentration range from 0.3 to 10 ng/ml. The calibration curve was prepared. The area of the peak was proportional to the concentration of the analyte. The coefficient of determination r^2^ for validation was found to be 0.9993 (Figure 1B). The mean recovery rate was found to be 37.2 ± 1.4%, and the recovery rate was sufficient to quantify Ang II in the determined calibration range. The detection limit was determined to be 0.1 ng/ml. The lower limit of quantification was determined to be 0.3 ng/ml. After the correction by the loss of each procedure, the level of iAng II in NRVMs was calculated to be 0.63 pmol/mg.

### Developing Cardiomyocytes Expressed Ang II Type 1 Receptor on the Cell Surface Membrane and Expressed Ang II Type 2 Receptor Predominately on the Nucleus and the Sarcoplasmic Reticulum

To examine the subcellular localization of AT_1_R and AT_2_R, immunostaining followed by confocal microscopy was used. AT_1_R and AT_2_R were found to be present in mESC-CMs. Since conventional antibodies against AT_1_R have been reported to be nonspecific, we constructed recombinant adenoviruses to express the AT_1_R fluorescent fusion protein (AT_1_R-YFP) to probe the subcellular localization of AT_1_R. AT_1_R-YFP was mainly expressed on the cell surface membrane of mESC-CMs (Figure 1D). On the other hand, immunostaining of AT_2_R revealed that AT_2_R was expressed in a unique, web-like pattern (Figures 1E,F). Multiple antibodies against AT_2_R were used and similar expression pattern of AT_2_R was obtained. Co-staining with the nuclear stain DAPI and with antibodies against SERCA2 (a cardiac-specific isoform of SERCA) revealed that AT_2_R was located on the nucleus (Figures 1E,F) and the SR (Figure 1F). In addition, the distribution of AT_1_R and AT_2_R in NRVMs was also investigated (Supplementary Figures S2A,B). The results were consistent with the expression patterns of AT_1_R and AT_2_R in mESC-CMs. Western blot analysis in NRVMs confirmed the quality of the AT_2_R antibody used in our study (Supplementary Figures S2C).

### Extracellular Ang II Regulated Action Potentials in a Transient Manner Through the Ang II Type 1 Receptor–Dependent Pathway

**FIGURE 2:**
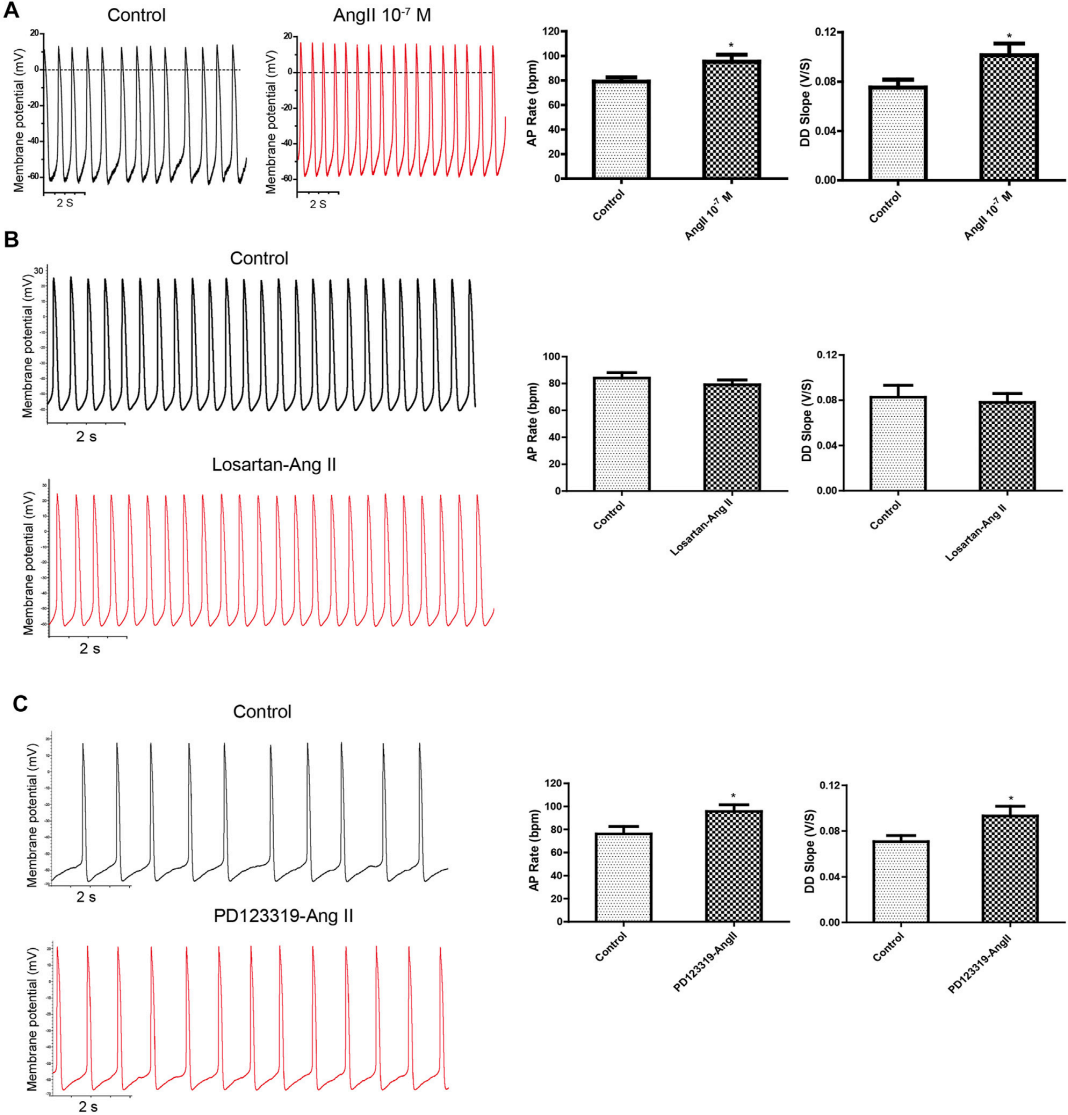
Application of exogenous Ang II increased APs transiently, and the effect could be attenuated by the AT_1_R blocker but not the AT_2_R blocker. **(A)** Ang II increased the APs of mESC-CMs in a transient manner. Raw traces of spontaneous APs at the basal level and upon treatment with 10^–7^ M Ang II in mESC-CMs **(left panel)**. Summarized data on the AP rate and the DD slope of APs **(right panel)**. **(B)** AT_1_R blocker attenuated Ang II–induced changes in APs. Raw traces of spontaneous APs at the basal level and upon treatment with 50 μM losartan, followed by the subsequent application of 10^–7^ M Ang II in mESC-CMs **(left panel)**. Summarized data on the AP rate and the DD slope of APs at the basal level or upon treatment with 50 μM losartan, followed by 10^–7^ M Ang II **(right panel)**. Losartan attenuated Ang II–induced changes in the parameters of APs. **(C)** AT_2_R blocker did not affect Ang II–induced changes in APs. Raw traces of spontaneous APs at the basal level and upon treatment with 10 μM PD123319, followed by the subsequent application of 10^–7^ M Ang II in mESC-CMs **(left panel)**. Summarized data on the AP rate and the DD slope of APs at the basal level or upon treatment with 10 μM PD123319, followed by 10^–7^ M Ang II **(right panel)**. PD123319 did not affect Ang II–induced changes in the parameters of APs. Values are mean ± SEM of 4–6 independent experiments. *p < 0.05 vs. control group.

The effect of exogenously applied Ang II on APs of mESC-CMs was first examined. When fluorescence-labeled Ang II was applied to mESC-CMs, Ang II was not observed to enter mESC-CMs even after being applied for 36 h (data not shown). The results hinted that exogenous Ang II may act on receptors located on the cell surface plasma membrane. Next, Ang II was applied to mESC-CMs to determine its effect on APs. Ang II (10^–7^ M) significantly and rapidly increased the AP rate and the DD slope of APs (Figure 2A), while it did not affect the action potential duration at 50% repolarization (APD_50_) and maximum diastolic depolarization (MDP) of APs in mESC-CMs (data not shown). These changes in the AP rate and the DD slope were transient (lasting 100–150 s after the addition of Ang II) and reversible, and they reappeared upon exposure to higher concentrations of Ang II. Furthermore, the changes in APs in response to Ang II (10^–7^ M) in the presence of AT_1_R blocker losartan (50 μM) or AT_2_R blocker PD123319 (10 μM) were examined. Preincubation of losartan attenuated the effects of Ang II on APs (Figure 2B), while preincubation of PD123319 did not affect the Ang II–induced response (Figure 2C). Similarly, preincubation of losartan attenuated the effects of Ang II on calcium transients (CaTs) (Supplementary Figures S3), while preincubation of PD123319 did not affect the Ang II–induced response (data not shown). These results suggested that AT_1_R, which is present on the cell surface plasma membrane of mESC-CMs, plays a critical role in mediating the transient effect of extracellular Ang II on APs and CaTs of mESC-CMs.

### Dual Current Patch Clamp Is an Advantageous Approach to Investigate the Effect of Intracellular Delivery of Drugs on the Action Potentials of Cardiomyocytes

**FIGURE 3:**
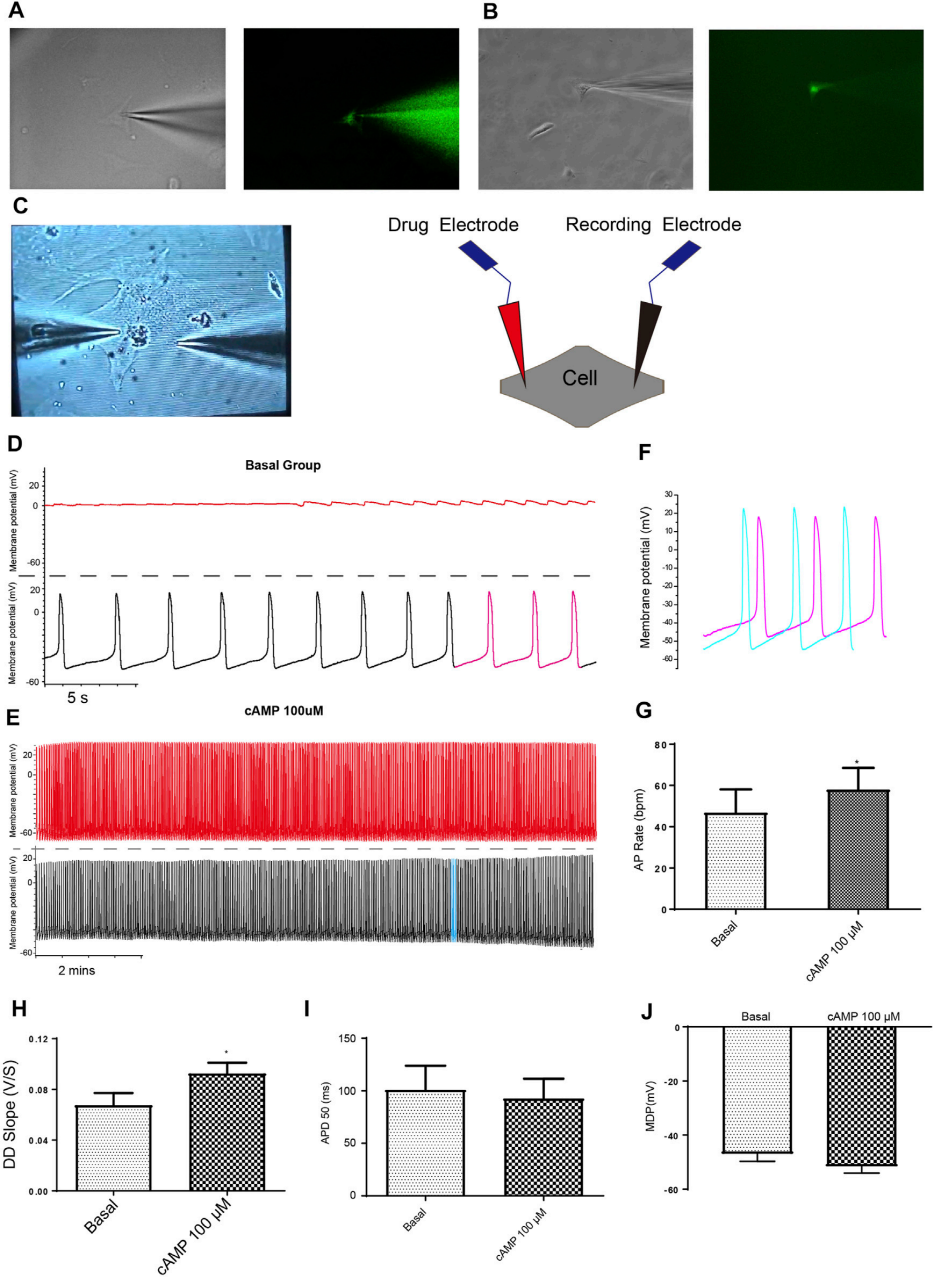
Dual current patch clamp is an advantageous approach to investigate the effect of intracellular delivery of drugs on the APs of mESC-CMs reliably. **(A)** Ang II-FITC can be successfully delivered into mESC-CMs using the glass electrode as shown by the appearance of the green fluorescence signal inside the cell. **(B)** Calcium indicator Fluo-4-IM can be delivered into mESC-CMs without a leak from the glass electrode. **(C)** Bright view of a dual current patch clamp experiment of mESC-CMs **(left panel)**. A schematic diagram on the dual patch clamp experimental configuration **(right panel)**. In dual patch clamp, AP measurement will first be made using the “recording electrode” [as shown by black traces in **(D)** and **(E)**; on the other hand, red traces in **(D)** and **(E)** represent recording in the “drug electrode”]. Before the membrane breakage by the “drug electrode”, the recording in the “recording electrode” serves as the “basal” level recording. After membrane breakage of the “drug electrode” and thereby the delivery of the drug to the intracellular environment, the AP measured in the “recording electrode” represents the effect of the drug. **(D, E)** cAMP was used as a positive control to show the utilization of dual patch clamp. **(D)** Representative trace showing the basal APs in mESC-CMs (before membrane breakage by the “drug electrode”). Upper panel represents recording in the “drug electrode” while the lower panel represents recording in the “recording electrode”. **(E)** Representative trace showing the APs after intracellular cAMP delivery (after membrane breakage by the “drug electrode”). Upper panel represents recording in the “drug electrode” while the lower panel represents recording in the “recording electrode”. **(F)** Traces labeled as pink in **(D)** (represents APs before intracellular cAMP delivery) and as blue in **(E)** (represents APs after intracellular cAMP delivery) are overlaid for comparison. **(G–J)** Summarized data on the **(G)** AP rate, **(H)** DD slope, **(I)** APD 50, and **(J)** MDP of APs upon treatment with cAMP (100 µM). cAMP increased the AP rate and the DD slope of APs. Values are mean ± SEM of 6–10 independent experiments. *p < 0.05 vs. control group.

Next, we confirmed and showed that the formation of a patch by the glass electrode, followed by subsequent breakage of the membrane, could be employed to effectively deliver drugs into CMs. Ang II-FITC could be successfully delivered into mESC-CMs using the glass electrode as shown by the appearance of the green fluorescence signal inside the cell without leakage into the extracellular space (Figure 3A). Moreover, Fluo-4-IM, a membrane-impermeant Ca^2+^ indicator, could also be successfully delivered into mESC-CMs and generated high fluorescence intensity by binding intracellular Ca^2+^ (Figure 3B).

Although a single glass electrode could simultaneously deliver drugs and record APs, basal APs before drug delivery (i.e., before rupture by whole-cell patch-clamp configuration) cannot be obtained. To circumvent this limitation, we developed a dual current patch clamp method (Figure 3C). It is known that intracellular cyclic AMP (cAMP) increases the rate of AP generation in the cardiac pacemaker cells. Therefore, cAMP was selected as a positive control to assess whether the dual current patch clamp is an efficient and reliable method to investigate the effect of intracellular delivery of drugs on the APs of CMs. As expected, cAMP markedly increased the AP rate and the DD slope without affecting the APD_50_ and MDP (Figures 3D–J). Thus, our results showed that dual current patch clamp is an advantageous approach to investigating the effect of intracellular delivery of drugs on the APs of CMs.

### Intracellular Ang II Decreased the Action Potentials of Developing Cardiomyocytes Through the Ang II Type 2 Receptor–Dependent Pathway

**FIGURE 4:**
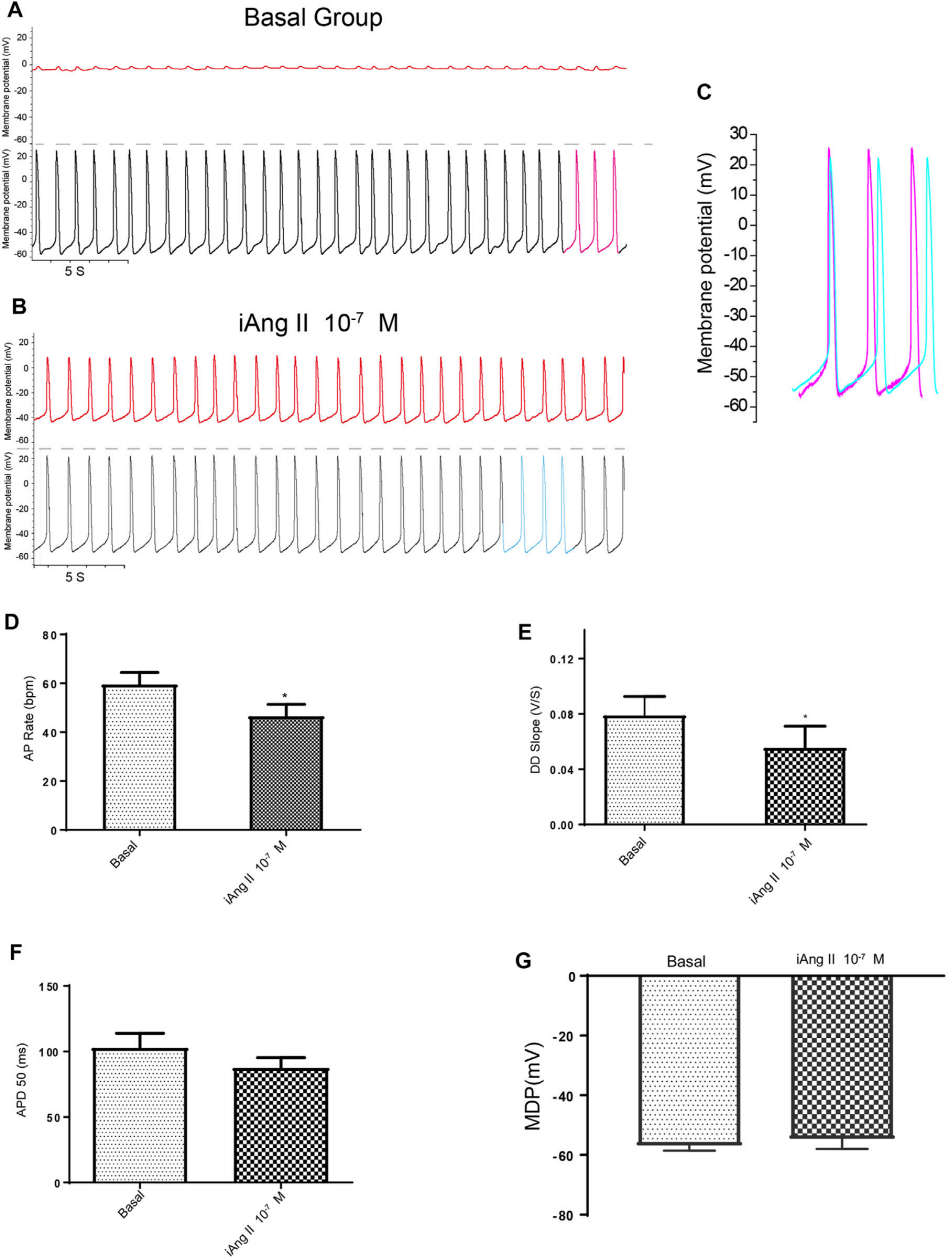
iAng II decreased the APs of mESC-CMs in a persistent manner. **(A)** Dual current patch clamp of APs of mESC-CMs without intracellular drug delivery (i.e., before membrane breakage by the “drug electrode”). Upper panel represents recording in the “drug electrode” while the lower panel represents recording in the “recording electrode.” **(B)** Dual current patch clamp of APs of mESC-CMs with intracellular delivery of Ang II (i.e., after membrane breakage by the “drug electrode”). Upper panel represents recording in the “drug electrode” while the lower panel represents recording in the “recording electrode.” **(C)** Traces labeled as pink in **(A)** (represents APs before iAng II delivery) and as blue in **(B)** (represents APs after iAng II delivery) are overlaid for comparison. **(D–G)** Summarized data on the **(D)** AP rate, **(E)** DD slope, **(F)** APD 50, and **(G)** MDP of APs upon treatment with iAng II. Values are mean ± SEM of 6–10 independent experiments. *p < 0.05 vs. control group.

**FIGURE 5:**
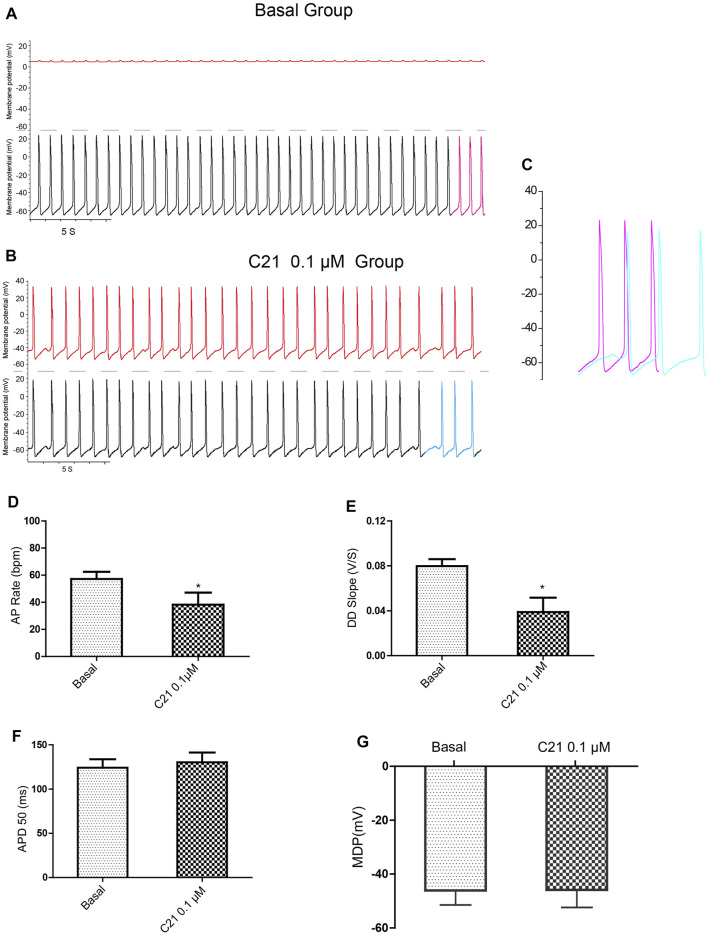
Intracellular delivery of AT_2_R activator C21 decreased the APs of mESC-CMs. **(A)** Dual current patch clamp of APs of mESC-CMs without intracellular drug delivery (i.e., before membrane breakage by the “drug electrode”). Upper panel represents recording in the “drug electrode” while the lower panel represents recording in the “recording electrode.” **(B)** Dual current patch clamp of APs of mESC-CMs with intracellular delivery of C21 (i.e., after membrane breakage by the “drug electrode”). Upper panel represents recording in the “drug electrode” while the lower panel represents recording in the “recording electrode.” **(C)** Traces labeled as pink in **(A)** (represents APs before intracellular C21 delivery) and as blue in **(B)** (represents APs after intracellular intracellular C21 delivery) are overlaid for comparison. **(D–G)** Summarized data on the **(D)** AP rate, **(E)** DD slope, **(F)** APD50, and **(G)** MDP of APs upon treatment with intracellular C21. Values are mean ± SEM of 6–10 independent experiments. *p < 0.05 vs. control group.

Using dual current patch clamp, Ang II was delivered intracellularly into mESC-CMs. iAng II (10^–7^ M) decreased the pacemaker activity of mESC-CMs (Figures 4A–C). iAng II reduced the AP rate (Figure 4D) and decreased the DD slope (Figure 4E) without affecting the APD_50_ (Figure 4F) and MDP (Figure 4G); intracellular delivery of the solvent did not exert any effect (Supplementary Figures S4). Unexpectedly, intracellular delivery of AT_1_R blocker losartan (50 μM) and AT_2_R blocker PD123319 (10 μM) alone did not affect the APs of mESC-CMs (Supplementary Figures S5, S6), hinting that endogenously produced Ang II in an unstimulated condition may not be present in significant amounts to activate the AT_1_R- or AT_2_R-dependent pathway. Interestingly, intracellular delivery of AT_2_R activator C21 (0.1 µM) (Wan et al., 2004) significantly decreased the APs of mESC-CMs (Figure 5), indicating that activation of AT_2_R would decrease APs. Similar results were obtained when NRVMs were used (Supplementary Figures S7, S8). Since a decent amount of iAng II and the activation of intracellular AT_2_R would similarly decrease the APs of developing CMs, our results suggested that iAng II activates intracellular AT_2_R, leading to a decrease in APs in CMs.

### Effect of Intracellular Ang II on the Automaticity of Mouse Embryonic Stem Cell–Derived Cardiomyocytes Was Associated With the Activity of Ryanodine Receptor Isoform 2

The data presented so far suggested that the iAng II signaling pathway is different from the signaling pathway of extracellular Ang II and indicated that iAng II exerts its effect predominantly through AT_2_R. Since AT_2_R is mainly located on the SR, we speculated that iAng II may decrease the APs by regulating the SR Ca^2+^ release channel RyR2. Caffeine can trigger Ca^2+^ release by reducing the threshold for luminal Ca^2+^ activation of RyR2, which is usually used to examine the activity of RyR2 (Kong et al., 2008). To investigate whether RyR2 contributes to the effects of iAng II on APs, we examined the changes in APs in response to the RyR2 activator caffeine in the presence of iAng II (10^–7^ M).

**FIGURE 6:**
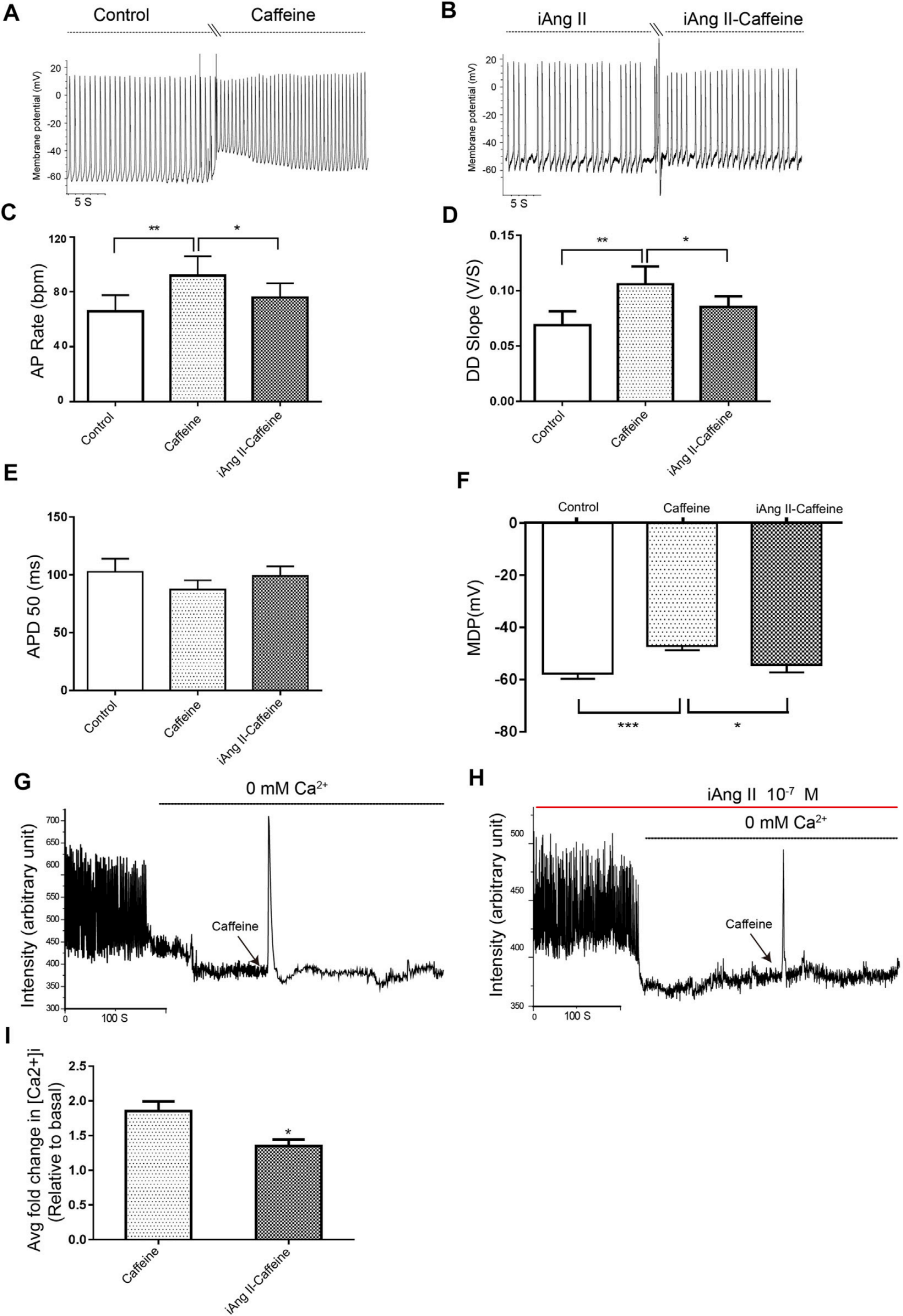
iAng II attenuated caffeine-induced changes of APs and caffeine-induced Ca^2+^ release in mESC-CMs. **(A)** Raw traces of spontaneous APs at the basal level and upon treatment with caffeine (5 μM) in mESC-CMs. **(B)** Raw traces of spontaneous APs upon treatment with iAng II (10^–7^ M) followed by the subsequent application of caffeine in mESC-CMs. **(C–F)** Summarized data on the **(C)** AP rate, **(D)** DD slope, **(E)** APD50, and **(F)** MDP upon treatment with caffeine (5 μM) alone or upon treatment with iAng II (10^–7^ M) followed by caffeine (5 μM). **(G)** Raw traces of changes in Fluo-4 fluorescence at the basal level and upon treatment with caffeine (5 μM) in mESC-CMs. **(H)** Raw traces of changes in Fluo-4 fluorescence with caffeine in the presence of iAng II (10^–7^ M) in mESC-CMs. **(I)** Summarized data on the effects of iAng II (10^–7^ M) on the caffeine-induced Ca^2+^ release in mESC-CMs when external Ca^2+^ was absent. Values are mean ± SEM of 6–10 independent experiments. *p < 0.05 vs. control group.

Application of iAng II partly attenuated the effects of caffeine on the automaticity of mESC-CMs (Figures 6A–F). Furthermore, in the absence of external Ca^2+^, iAng II (10^–7^ M) reduced the caffeine-induced Ca^2+^ release in mESC-CMs (Figures 6G–I). These results suggested that iAng II negatively regulates the automaticity of mESC-CMs through inhibiting the activity of RyR2.

## Discussion

The present study provides significant new insights into the role of extracellular and intracellular Ang II in the regulation of automaticity of CMs. Our main findings include the following: 1) iAng II could be detected in both NRVMs and mESC-CMs, 2) AT_1_R is located on the plasma membrane, while AT_2_R is predominately located on the nucleus and the SR of developing CMs, 3) extracellular Ang II increases APs in a transient manner through the AT_1_R-dependent pathway, 4) iAng II decreases APs in a persistent manner through the AT_2_R-dependent pathway, and 5) iAng II regulates the automaticity of developing CMs by decreasing the activity of RyR2.

### Intracellular Ang II Can Be Detected in Developing Cardiomyocytes

Increasing evidence revealed that Ang II may function as an intracellular peptide to activate intracellular/nuclear receptors and subsequently activates downstream signaling effectors independent of cell surface receptors in CMs. However, previous studies focused on the iAng II function in CMs were restricted to nonspecific detection approaches because of the lack of the specific Ang II antibody and its low concentration in CMs. In our study, we first built up a detection system (including sample collection, purification, concentration, and detection by LC-MS) to detect and to quantify the iAng II in developing CMs. Our results showed that the concentration of iAng II in NRVMs was 0.63 pmol/mg, which was similar to that detected by ELISA in rat CMs (Singh et al., 2008).

Previous reports suggested that Ang II can be produced endogenously in different cell types, including adult CMs (Baker et al., 1992; Malhotra et al., 1999; Paul et al., 2006; Singh et al., 2007; Tsai et al., 2008). However, whether Ang II can be produced locally in early developing CMs and, if yes, what its role is in regulating the unique characteristic automaticity of early developing CMs and sinoatrial cells are unknown. Our results revealed that endogenous Ang II is present in mESC-CMs. This endogenous Ang II is unlikely to be attributed to the entry of extracellular Ang II, as the uptake of exogenously applied fluorescently labeled Ang II by mESC-CMs was not observed after 36 h of application. This result indicated that mESC-CMs can probably synthesize their own Ang II.

In addition to synthesizing their own Ang II, the two distinct subtypes of Ang II receptors, AT_1_R and AT_2_R, were both found to be present in the developing CMs; AT_1_R is present on the cell surface membrane, while AT_2_R is present on the nucleus and the SR. After decades of research, many components of the RAS, including angiotensinogen, angiotensin converting enzyme, Ang II, and Ang II receptors, have been detected in the whole heart where myocytes and non-myocytes are present (Serneri et al., 2001; van Kats et al., 2001; Danser, 2003). However, it is unclear whether these components are synthesized locally in the CMs. For instance, the origin of renin, which is required for the generation of local Ang II, is still unclear. Therefore, the exact synthesis mechanism for the iAng II in CMs has remained an unanswered question. We speculate that the local Ang II production in developing CMs may be the result of a combination of RAS component uptake from the extracellular environment, local synthesis of the RAS components, and in-site synthesis of iAng II. On the other hand, while it was previously technically challenging to detect genuine iAng II due to the potentially nonspecific method and its extremely low intracellular level, the UHPLC-ESI-MS/MS method developed in our current study has solidly proven the existence of iAng II in developing CMs.

### Functional Intracellular Ang II–Ang II Type 2 Receptor–Ryanodine Receptor Isoform 2 Pathway Is Present in Cardiomyocytes, Counteracting the Effect of Extracellular Ang II in Regulating the Automaticity of Cardiomyocytes

With components of local RAS being present, Ang II shall regulate some of the functions of these developing CMs. Previous studies have shown that extracellular Ang II increased the amplitude of CaTs and the contraction amplitude in paced ESC-CMs or in embryoid bodies containing CMs (Sedan et al., 2008; Lagerqvist et al., 2011). Automaticity is the unique feature of developing CMs. Our study showed that extracellular Ang II increased the AP rate and the DD slope of the developing CMs through the AT_1_R-dependent pathway. Importantly, our study revealed that iAng II that is produced endogenously would exert the opposite effect on spontaneous APs; iAng II decreased both the AP rate and the DD slope of APs. Interestingly, our results showed that intracellular delivery of AT_2_R activator C21 exerted a similar effect to that of iAng II, suggesting that iAng II acts through AT_2_R. In addition, iAng II attenuated the effect of the activation of RyR2 and decreased the Ca^2+^ released from the RyR2. Previous studies on automaticity of sinoatrial nodal cells showed that a decrease in RyR2 activity would decrease the AP rate (Rigg et al., 2000). Our novel results suggest that iAng II decreases the activity of RyR2, leading to the decrease in APs.

**FIGURE 7:**
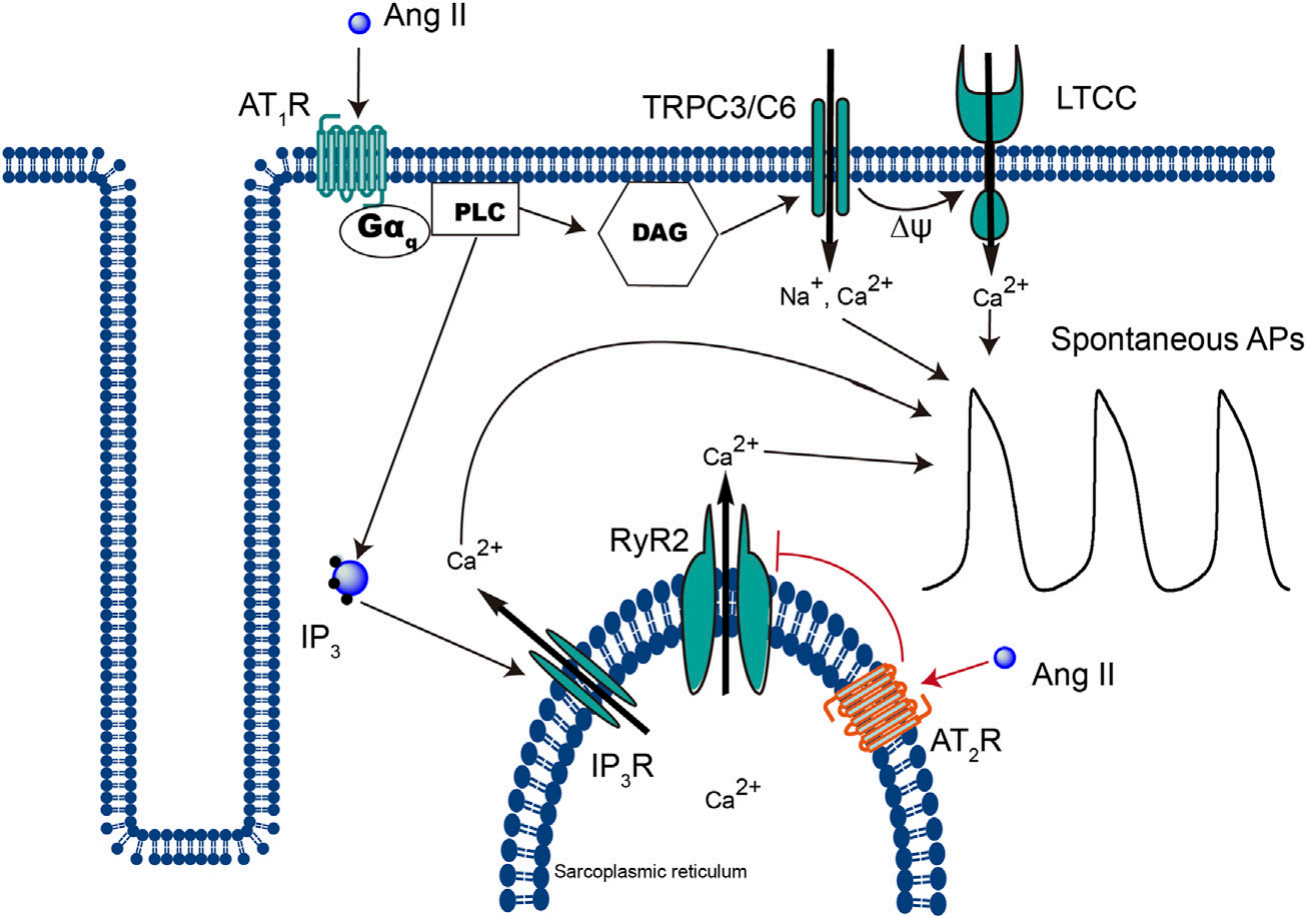
Schematic representation of the different signaling pathways that extracellular Ang II and iAng II utilize to regulate the automaticity of CMs. Extracellular Ang II, upon binding to AT_1_R, leads to the production of DAG and IP_3_, which open TRPC3/C6 and subsequently LTCC and IP_3_R, respectively, and increases the automaticity of CMs. On the other hand, binding of iAng II to AT_2_R decreases the automaticity of CMs by reducing the activity of RyR2.

AT_1_R is a typical seven-transmembrane GPCR. It was widely reported that the majority of extracellular Ang II–evoked cellular responses are mediated through the activation of the “classical” signaling mechanism (George et al., 2010; Savoia et al., 2011) (Figure 7). On the contrary, the role of iAng II, the identity of AT_2_R, and the signaling pathway through which iAng II/AT_2_R mediate their effect are still under extensive investigation.

Several previous reports have shown that iAng II implemented its effects *via* AT_2_R (Tadevosyan et al., 2015; Zhao et al., 2015). In CMs, iAng II has been shown to interact with nuclear AT_2_R and regulate RNA synthesis, cell proliferation, and collagen secretion (Singh et al., 2008). Our results suggest that iAng II would act through AT_2_R and negatively regulate automaticity on a much shorter timescale.

On the other hand, our results indicated that iAng II negatively regulates RyR2 and controls APs in CMs. Since iAng II also concomitantly mediates its effect *via* AT_2_R, it is reasonable to speculate that binding of iAng II to AT_2_R would decrease the activity of RyR2. However, whether iAng II/AT_2_R can directly couple to RyR2 and downregulate its activity, or whether further downstream signaling is involved, is unknown. Interestingly, AT_2_R was first described as a thiol-potentiated Ang II receptor in 1989. Previous studies reported that AT_2_R may exist in the nuclear membrane and the mitochondrial inner membrane. However, in comparison to AT_1_R, the functions of AT_2_R are relatively unexplored. Some studies reported that AT_2_R can increase nitric oxide (NO) release, reduce vascular tone, and attenuate ischemia reperfusion injury in the myocardium (Mendoza-Torres et al., 2018; Paz Ocaranza et al., 2020). NO has a function to diminish the opening probability of RyR2, which is a mechanism that could be cardioprotective (Lim et al., 2008). Thus, the pathway AT_2_R-NO-RyR2 may be a potential mechanism by which AT_2_R inhibits RyR2. Further studies would be needed to investigate how AT_2_R can modulate the activity of RyR2.

While at a physiological level, it has been known for a long time that an increase in the intracellular Ca^2+^ level potentiates the activity of RyR2 (in the classical Ca^2+^-induced Ca^2+^ release mechanism) and that at the pharmacological level, the activators and inhibitors of RyR2 are well studied, knowledge about physiological inhibitors of RyR2 remains scarce. In our present study, we found that iAng II, probably by activating AT_2_R at the SR, decreased the Ca^2+^ release from RyR2. However, whether iAng II/AT_2_R can directly couple to RyR2 and downregulate its activity, or whether further downstream signaling is involved, is unknown. Further study shall concentrate on elucidating the mechanism behind this potentially important signaling pathway (Figure 7).

Some previous reports have documented that activation of AT_1_R and AT_2_R would lead to the opposite effect in the cardiovascular system (Jones et al., 2008; Padia and Carey, 2013; Lagatta et al., 2018); however, there is no study clearly documenting whether extracellular Ang II and iAng II would exert an opposing effect or not. Our study is the first study clearly demonstrating that extracellular Ang II and iAng II activate AT_1_R and AT_2_R, respectively, to exert opposing cellular functions.

## Conclusion

In conclusion, our study has invented a novel UHPLC-ESI-MS/MS method to solidly reveal the existence of iAng II in developing CMs. Our study has also shown that AT_1_R is located on the plasma membrane, while AT_2_R is predominately located on the nucleus and the SR of developing CMs. In addition, our study revealed that extracellular Ang II regulates APs through AT_1_R in a transient manner. Importantly, by employing the novel and technically challenging dual patch clamp technique and the calcium imaging technique, we clearly showed that iAng II negatively regulates the automaticity of developing CMs through the AT_2_R-RyR2 pathway. The present investigation has uncovered the vital role of iAng II and provided the potential mechanisms of how Ang II regulates cardiac automaticity.

## Data Availability

The original contributions presented in the study are included in the article/Supplementary Material; further inquiries can be directed to the corresponding author.

## References

[B1] Akazawa H. Yano M. Yabumoto C. Kudo-Sakamoto Y. Komuro I. (2013). Angiotensin II Type 1 and Type 2 Receptor-Induced Cell Signaling. Cpd 19 (17), 2988–2995. 10.2174/1381612811319170003 23176210

[B2] Asada H. Horita S. Hirata K. Shiroishi M. Shiimura Y. Iwanari H. (2018). Crystal Structure of the Human Angiotensin II Type 2 Receptor Bound to an Angiotensin II Analog. Nat. Struct. Mol. Biol. 25 (7), 570–576. 10.1038/s41594-018-0079-8 29967536

[B3] Baker K. M. Booz G. W. Dostal D. E. (1992). Cardiac Actions of Angiotensin II: Role of an Intracardiac Renin-Angiotensin System. Annu. Rev. Physiol. 54, 227–241. 10.1146/annurev.ph.54.030192.001303 1562174

[B4] Cheng H. Lederer W. J. (2008). Calcium sparks. Physiol. Rev. 88 (4), 1491–1545. 10.1152/physrev.00030.2007 18923188

[B5] Danser A. H. (2003). Local Renin-Angiotensin Systems: the Unanswered Questions. Int. J. Biochem. Cel Biol 35 (6), 759–768. 10.1016/s1357-2725(02)00178-4 12676161

[B6] Danser A. Saris J. J. Schuijt M. P. van Kats J. P. (1999). Is There a Local Renin-Angiotensin System in the Heart?. Cardiovasc. Res. 44 (2), 252–265. 10.1016/S0008-6363(99)00202-3 10690302

[B7] de Gasparo M. Catt K. J. Inagami T. Wright J. W. Unger T. (2000). International union of Pharmacology. XXIII. The Angiotensin II Receptors. Pharmacol. Rev. 52, 415–472. 10977869

[B8] De Mello W. C. (2015). Intracellular Angiotensin II Disrupts Chemical Communication and Impairs Metabolic Cooperation between Cardiac Myocytes. Peptides 72, 57–60. 10.1016/j.peptides.2015.04.001 25882009

[B9] Gallinat S. Busche S. Schütze S. Krönke M. Unger T. (1999). AT2 Receptor Stimulation Induces Generation of Ceramides in PC12W Cells. Febs Lett. 443 (1), 75–79. 10.1016/s0014-5793(98)01675-5 9928956

[B10] George A. J. Thomas W. G. Hannan R. D. (2010). The Renin-Angiotensin System and Cancer: Old Dog, New Tricks. Nat. Rev. Cancer 10 (11), 745–759. 10.1038/nrc2945 20966920

[B11] Harada K. Sugaya T. Murakami K. Yazaki Y. Komuro I. (1999). Angiotensin II Type 1A Receptor Knockout Mice Display Less Left Ventricular Remodeling and Improved Survival after Myocardial Infarction. Circulation 100 (20), 2093–2099. 10.1161/01.Cir.100.20.2093 10562266

[B12] Inuzuka T. Fujioka Y. Tsuda M. Fujioka M. Satoh A. O. Horiuchi K. (2016). Attenuation of Ligand-Induced Activation of Angiotensin II Type 1 Receptor Signaling by the Type 2 Receptor via Protein Kinase C. Sci. Rep. 6. 10.1038/srep21613 PMC4746669 26857745

[B13] Jones E. S. Vinh A. McCarthy C. A. Gaspari T. A. Widdop R. E. (2008). AT2 Receptors: Functional Relevance in Cardiovascular Disease. Pharmacol. Ther. 120 (3), 292–316. 10.1016/j.pharmthera.2008.08.009 18804122 PMC7112668

[B14] Karnik S. S. Unal H. Kemp J. R. Tirupula K. C. Eguchi S. Vanderheyden P. M. L. (2015). International Union of Basic and Clinical Pharmacology. XCIX. Angiotensin Receptors: Interpreters of Pathophysiological Angiotensinergic Stimuli. Pharmacol. Rev. 67 (4), 754–819. 10.1124/pr.114.010454 26315714 PMC4630565

[B15] Kong H. Jones P. P. Koop A. Zhang L. Duff H. J. Chen S. R. W. (2008). Caffeine Induces Ca2+ Release by Reducing the Threshold for Luminal Ca2+ Activation of the Ryanodine Receptor. Biochem. J. 414 (3), 441–452. 10.1042/BJ20080489 18518861 PMC2747660

[B16] Lagatta D. C. Kuntze L. B. Ferreira-Junior N. C. Resstel L. B. M. (2018). Medial Prefrontal Cortex TRPV1 and CB1 Receptors Modulate Cardiac Baroreflex Activity by Regulating the NMDA Receptor/nitric Oxide Pathway. Pflugers Arch. - Eur. J. Physiol. 470 (10), 1521–1542. 10.1007/s00424-018-2149-5 29845313

[B17] Lagerqvist E. L. Finnin B. A. Pouton C. W. Haynes J. M. (2011). Endothelin-1 and Angiotensin II Modulate Rate and Contraction Amplitude in a Subpopulation of Mouse Embryonic Stem Cell-Derived Cardiomyocyte-Containing Bodies. Stem Cel Res. 6 (1), 23–33. 10.1016/j.scr.2010.09.001 20970401

[B18] Lakatta E. G. Maltsev V. A. Vinogradova T. M. (2010). A Coupled SYSTEM of Intracellular Ca 2+ Clocks and Surface Membrane Voltage Clocks Controls the Timekeeping Mechanism of the Heart's Pacemaker. Circ. Res. 106 (4), 659–673. 10.1161/CIRCRESAHA.109.206078 20203315 PMC2837285

[B19] Law S. K. Leung C. S.-L. Yau K. L. Tse C. L. Wong C. K. Leung F. P. (2013). Regulation of Multiple Transcription Factors by Reactive Oxygen Species and Effects of Pro-inflammatory Cytokines Released during Myocardial Infarction on Cardiac Differentiation of Embryonic Stem Cells. Int. J. Cardiol. 168 (4), 3458–3472. 10.1016/j.ijcard.2013.04.178 23706318

[B20] Lim G. Venetucci L. Eisner D. A. Casadei B. (2008). Does Nitric Oxide Modulate Cardiac Ryanodine Receptor Function? Implications for Excitation-Contraction Coupling. Cardiovasc. Res. 77 (2), 256–264. 10.1093/cvr/cvm012 18006480

[B21] Lokuta A. J. Cooper C. Gaa S. T. Wang H. E. Rogers T. B. (1994). Angiotensin II Stimulates the Release of Phospholipid-Derived Second Messengers through Multiple Receptor Subtypes in Heart Cells. J. Biol. Chem. 269 (7), 4832–4838. 10.1016/S0021-9258(17)37619-6 8106454

[B22] Malhotra R. Sadoshima J. Brosius F. C. Izumo S. (1999). Mechanical Stretch and Angiotensin II Differentially Upregulate the Renin-Angiotensin System in Cardiac Myocytes *In Vitro* . Circ. Res. 85 (2), 137–146. 10.1161/01.Res.85.2.137 10417395

[B23] Mendoza-Torres E. Riquelme J. A. Vielma A. Sagredo A. R. Gabrielli L. Bravo-Sagua R. (2018). Protection of the Myocardium against Ischemia/reperfusion Injury by Angiotensin-(1-9) through an AT2R and Akt-dependent Mechanism. Pharmacol. Res. 135, 112–121. 10.1016/j.phrs.2018.07.022 30048754

[B24] Monfredi O. Maltsev V. A. Lakatta E. G. (2013). Modern Concepts Concerning the Origin of the Heartbeat. Physiology 28 (2), 74–92. 10.1152/physiol.00054.2012 23455768 PMC3768086

[B25] Muller J. Corodimas K. P. Fridel Z. LeDoux J. E. (1997). Functional Inactivation of the Lateral and Basal Nuclei of the Amygdala by Muscimol Infusion Prevents Fear Conditioning to an Explicit Conditioned Stimulus and to Contextual Stimuli. Behav. Neurosci. 111 (4), 683–691. 10.1037/0735-7044.111.4.683 9267646

[B26] Ng S.-Y. Chin C.-H. Lau Y.-T. Luo J. Wong C.-K. Bian Z.-X. (2010). Role of Voltage-Gated Potassium Channels in the Fate Determination of Embryonic Stem Cells. J. Cel. Physiol. 224 (1), a–n. 10.1002/jcp.22113 20333647

[B27] Onohara N. Nishida M. Inoue R. Kobayashi H. Sumimoto H. Sato Y. (2006). TRPC3 and TRPC6 Are Essential for Angiotensin II-Induced Cardiac Hypertrophy. Embo J. 25 (22), 5305–5316. 10.1038/sj.emboj.7601417 17082763 PMC1636614

[B28] Padia S. H. Carey R. M. (2013). AT2 Receptors: Beneficial Counter-regulatory Role in Cardiovascular and Renal Function. Pflugers Arch. - Eur. J. Physiol. 465 (1), 99–110. 10.1007/s00424-012-1146-3 22949090 PMC3548020

[B29] Paul M. Poyan Mehr A. Kreutz R. (2006). Physiology of Local Renin-Angiotensin Systems. Physiol. Rev. 86 (3), 747–803. 10.1152/physrev.00036.2005 16816138

[B30] Paz Ocaranza M. Riquelme J. A. García L. Jalil J. E. Chiong M. Santos R. A. S. (2020). Counter-regulatory Renin-Angiotensin System in Cardiovascular Disease. Nat. Rev. Cardiol. 17 (2), 116–129. 10.1038/s41569-019-0244-8 31427727 PMC7097090

[B31] Qi Z. Wong C. K. Suen C. H. Wang J. Long C. Sauer H. (2016). TRPC3 Regulates the Automaticity of Embryonic Stem Cell-Derived Cardiomyocytes. Int. J. Cardiol. 203, 169–181. 10.1016/j.ijcard.2015.10.018 26512833

[B32] Rigg L. Heath B. M. Cui Y. Terrar D. A. (2000). Localisation and Functional Significance of Ryanodine Receptors during β-adrenoceptor Stimulation in the guinea-pig Sino-Atrial Node. Cardiovasc. Res. 48 (2), 254–264. 10.1016/s0008-6363(00)00153-x 11054472

[B33] Savoia C. Burger D. Nishigaki N. Montezano A. Touyz R. M. (2011). Angiotensin II and the Vascular Phenotype in Hypertension. Expert Rev. Mol. Med. 13. 10.1017/S1462399411001815 21450123

[B34] Sedan O. Dolnikov K. Zeevi-Levin N. Leibovich N. Amit M. Itskovitz-Eldor J. (2008). 1,4,5-Inositol Trisphosphate-Operated Intracellular Ca2+Stores and Angiotensin-II/Endothelin-1 Signaling Pathway Are Functional in Human Embryonic Stem Cell-Derived Cardiomyocytes. Stem Cells 26 (12), 3130–3138. 10.1634/stemcells.2008-0777 18818435

[B35] Senbonmatsu T. Saito T. Landon E. J. Watanabe O. Price E. Roberts R. L. (2003). A Novel Angiotensin II Type 2 Receptor Signaling Pathway: Possible Role in Cardiac Hypertrophy. EMBO J. 22 (24), 6471–6482. 10.1093/emboj/cdg637 14657020 PMC291832

[B36] Serneri G. G. N. Boddi M. Cecioni I. Vanni S. Coppo M. Papa M. L. (2001). Cardiac Angiotensin II Formation in the Clinical Course of Heart Failure and its Relationship with Left Ventricular Function. Circ. Res. 88 (9), 961–968. 10.1161/hh0901.089882 11349007

[B37] Singh V. P. Le B. Bhat V. B. Baker K. M. Kumar R. (2007). High-glucose-induced Regulation of Intracellular ANG II Synthesis and Nuclear Redistribution in Cardiac Myocytes. Am. J. Physiology-Heart Circulatory Physiol. 293 (2), H939–H948. 10.1152/ajpheart.00391.2007 17483239

[B38] Singh V. P. Le B. Khode R. Baker K. M. Kumar R. (2008). Intracellular Angiotensin II Production in Diabetic Rats Is Correlated with Cardiomyocyte Apoptosis, Oxidative Stress, and Cardiac Fibrosis. Diabetes 57 (12), 3297–3306. 10.2337/db08-0805 18829990 PMC2584136

[B39] Tadevosyan A. Létourneau M. Folch B. Doucet N. Villeneuve L. R. Mamarbachi A. M. (2015). Photoreleasable Ligands to Study Intracrine Angiotensin II Signalling. J. Physiol. 593 (3), 521–539. 10.1113/jphysiol.2014.279109 25433071 PMC4324703

[B40] Tadevosyan A. Maguy A. Villeneuve L. R. Babin J. Bonnefoy A. Allen B. G. (2010). Nuclear-delimited Angiotensin Receptor-Mediated Signaling Regulates Cardiomyocyte Gene Expression. J. Biol. Chem. 285 (29), 22338–22349. 10.1074/jbc.M110.121749 20463030 PMC2903375

[B41] Tadevosyan A. Xiao J. Surinkaew S. Naud P. Merlen C. Harada M. (2017). Intracellular Angiotensin‐II Interacts with Nuclear Angiotensin Receptors in Cardiac Fibroblasts and Regulates RNA Synthesis, Cell Proliferation, and Collagen Secretion. Jaha 6 (4). 10.1161/jaha.116.004965 PMC5533010 28381466

[B42] Thomas W. G. Mendelsohn F. A. O. (2003). Angiotensin Receptors: Form and Function and Distribution. Int. J. Biochem. Cel Biol. 35 (6), 774–779. 10.1016/s1357-2725(02)00263-7 12676163

[B43] Tsai C.-T. Lai L.-P. Hwang J.-J. Chen W.-P. Chiang F.-T. Hsu K.-L. (2008). Renin-angiotensin System Component Expression in the HL-1 Atrial Cell Line and in a Pig Model of Atrial Fibrillation. J. Hypertens. 26 (3), 570–582. 10.1097/HJH.0b013e3282f34a4a 18300870

[B44] van Kats J. P. Methot D. Paradis P. Silversides D. W. Reudelhuber T. L. (2001). Use of a Biological Peptide Pump to Study Chronic Peptide Hormone Action in Transgenic Mice. J. Biol. Chem. 276 (47), 44012–44017. 10.1074/jbc.M106132200 11551931

[B45] Wan Y. Wallinder C. Plouffe B. Beaudry H. Mahalingam A. K. Wu X. (2004). Design, Synthesis, and Biological Evaluation of the First Selective Nonpeptide AT2 Receptor Agonist. J. Med. Chem. 47 (24), 5995–6008. 10.1021/jm049715t 15537354

[B46] Wollert K. C. Taga T. Saito M. Narazaki M. Kishimoto T. Glembotski C. C. (1996). Cardiotrophin-1 Activates a Distinct Form of Cardiac Muscle Cell Hypertrophy. J. Biol. Chem. 271 (16), 9535–9545. 10.1074/jbc.271.16.9535 8621626

[B47] Wong C.-K. So W.-Y. Law S.-K. Leung F.-P. Yau K.-L. Yao X. (2012). Estrogen Controls Embryonic Stem Cell Proliferation via Store-Operated Calcium Entry and the Nuclear Factor of Activated T-Cells (NFAT). J. Cel. Physiol. 227 (6), 2519–2530. 10.1002/jcp.22990 21898397

[B48] Yusuf S. Yusuf S. Sleight P. Pogue J. Bosch J. Davies R. (2000). Effects of an Angiotensin-Converting-Enzyme Inhibitor, Ramipril, on Cardiovascular Events in High-Risk Patients. N. Engl. J. Med. 342 (3), 145–153. 10.1056/nejm200001203420301 10639539

[B49] Zaman M. A. Oparil S. Calhoun D. A. (2002). Drugs Targeting the Renin-Angiotensin-Aldosterone System. Nat. Rev. Drug Discov. 1 (8), 621–636. 10.1038/nrd873 12402502

[B50] Zhao Y. Lützen U. Fritsch J. Zuhayra M. Schütze S. Steckelings U. M. (2015). Activation of Intracellular Angiotensin AT2 Receptors Induces Rapid Cell Death in Human Uterine Leiomyosarcoma Cells. Clin. Sci. 128 (9), 567–578. 10.1042/cs20140627 25487516

