# Supplementary material for: Extracellular and Intracellular Angiotensin II Regulate the Automaticity of Developing Cardiomyocytes via Different Signaling Pathways
Source: Front Mol Biosci. 2021 Aug 25;8:699827. doi: 10.3389/fmolb.2021.699827 (PMC8425478; doi:10.3389/fmolb.2021.699827)
Supplement: Supplementary file 1 [file DataSheet1.docx]

**Extracellular and intracellular angiotensin II regulate the automaticity of** **developing cardiomyocytes via different signaling pathways**

Zenghua Qi ^1,2^, Tao Wang ^3^, Xiangmao Chen ^4^, Chun Kit Wong ^1^, Qianqian Ding ^1^, Heinrich Sauer ^6^, Zhi-Feng Chen^2^, Cheng Long ^4^, Xiaoqiang Yao ^5^, Zongwei Cai ^3^, Suk Ying Tsang ^1,7,8,#^

^1^ School of Life Sciences, The Chinese University of Hong Kong, Hong Kong SAR

^2^ Institute of Environmental Health and Pollution Control, School of Environmental Science and Engineering, Guangdong University of Technology, Guangzhou, Guangdong, China

^3^ State Key Laboratory of Environmental and Biological Analysis, Department of Chemistry, Hong Kong Baptist University, Hong Kong, China

^4^ School of Life Sciences, South China Normal University, Guangzhou, PR China

^5^ School of Biomedical Sciences, The Chinese University of Hong Kong, Hong Kong SAR

^6^ Department of Physiology, Justus Liebig University Giessen, Germany

^7^ Key Laboratory for Regenerative Medicine, Ministry of Education, The Chinese University of Hong Kong, Hong Kong SAR

^8^ State Key Laboratory of Agrobiotechnology, The Chinese University of Hong Kong, Hong Kong SAR

**SUPPLEMENTARY MATERIALS AND METHODS**

**1. Primary mouse embryonic fibroblast (MEF) culture**

This study was approved by the Animal Ethics Committee, the Chinese University of Hong Kong and conformed to Guide for the Care and Use of Laboratory Animals published by the United States National Institutes of Health (NIH Publication No. 80-23, revised 2011). MEFs were prepared from mouse embryos at 13-14 days of gestation. Pregnant CD-1 mice were euthanized by CO_2_ inhalation. Mouse embryos were collected. MEFs were prepared from mouse embryos using standard protocol. Briefly, the embryos were minced with dissecting scissors. Thereafter, the tissues were digested with 0.05% trypsin (Invitrogen) at 37 ^o^C for 30 minutes. Single MEFs were cultured in MEFs medium which contained DMEM (Invitrogen) supplemented with 15 % heat inactivated FBS (Invitrogen), 2 mM L-glutamine, 0.1 mM non-essential amino acids, 1 % v/v Penicillin-Streptomycin for 24 hours. Media was refreshed after 24 hours and the primary culture of MEFs was continued until the flask reached 90% confluency. MEFs were then harvested, freezed and stored in liquid nitrogen for further use.

**2. Mouse embryonic stem cell (mESC) culture**

mESC cell line D3 (ATCC, Manassas, VA, USA) was cultured on irradiated MEFs in undifferentiation medium which contained Dulbecco’s modified Eagle’s medium (DMEM) (Invitrogen, Carlsbad, CA, USA) supplemented with 15 % heat inactivated (57 ^o^C, 30 mins) fetal bovine serum (FBS) (Hyclone, GE Healthcare, South Logan, UT, USA), 0.1 mM β-mercaptoethanol (Sigma, St. Louis, MO, USA), 2 mM L-glutamine (Invitrogen), 0.1 mM non-essential amino acids (Invitrogen), 1 % v/v Penicillin-Streptomycin (Invitrogen) and 1,000 U/mL leukemia inhibitory factor (LIF) (Chemicon, Millipore, Billerica, MA, USA). Undifferentiated mESCs were split every 3 days at a density of 0.8×10^5^ cells per well of 6-well culture plate using 0.05% trypsin.

**3. Differentiation of mESCs to embryoid bodies (EBs) using hanging drop method**

Differentiation of mESCs was performed by hanging drop method. Briefly, on the day of differentiation, mESCs were dissociated with 0.05 % trypsin and resuspended in normal differentiation medium which had the same components as the undifferentiation medium except without LIF. EBs were formed in hanging drop by making each drop with 800 mESCs in 20 µL. mESCs started to differentiate from this day 0. On day 2, EBs were washed into 10-cm bacterial-grade dish with 10 mL differentiation medium. EBs remained in suspension state until day 7. EBs were seeded on 0.1% gelatin (Sigma)-coated culture dishes on day 7. Normally, spontaneously beating EBs, which represents the appearance of CMs, could be seen after the attachment of EBs.

**4. Isolation of mESC-CMs**

Embryoid bodies (EB) dissection and CM isolation were performed using method from previous published study [1]. The isolation was done 2 days before calcium imaging. Beating regions in EB were dissected using a 1 mL syringe with 27-G needle under dissection microscope. Dissected regions were placed in sterile 1.5 mL eppendorf with differentiation medium. The dissected regions were centrifuged at 1000rpm, 4 ^o^C for 5 minutes. Then, medium was removed and cell pellet was washed with PBS. Pellet was centrifuged at 1000rpm, 4 ^o^C for 5 minutes and PBS was removed. 1 mg collagenase B (Roche Diagnostics, Basel, Switzerland) was dissolved in 1 mL digestion solution (NB solution) which contained 120.0 mM NaCl, 5.4 mM KCl, 5.0 mM MgSO_4_, 5.0 mM Na Pyruvate, 20.0 mM glucose, 20.0 mM taurine, 10.0 mM HEPES and 30.0 μM CaCl_2_, pH adjusted to 6.9 by NaOH. Digestion solution with 1 mg/mL collagenase B was filtered through a 0.22 μM filter before adding to cells. The whole mixture was incubated at 37^o^C air shaker shaking at 150rpm for 30 minutes. After shaking, cells were centrifuged at 3000rpm, 4^o^C for 5 minutes and the digestion solution was removed. KB solution, which contained 85.0 mM KCl, 30.0 mM K_2_HPO4, 5.0 mM MgSO_4_, 1.0 mM EGTA, 5.0 mM pyruvic acid, 5.0 mM creatine, 20.0 mM taurine, 20.0 mM glucose, 87 µM of Na-ATP (Sigma), pH adjusted to 7.2 by NaOH, was used to resuspend the cell pellet for recovery of cells. Na-ATP was added immediately before use. Cells were incubated at room temperature and shaked at 600 rpm for 1 hour. After recovery, cells were seeded on glass slides pre-coated with 20 μg/mL laminin (Invitrogen, Carlsbad, CA, USA) in 0.1 % gelatin in KB solution for 40 minutes in 37 ^o^C, 5 % CO_2_ incubator. Thereafter, normal differentiation medium was added to the isolated cells for subsequent culture.

**5. Total RNA isolation and reverse transcription**

The liver was collected from the mouse and immediately ground under liquid nitrogen and stored at -80 °C until used for RNA preparation. Total RNA was isolated using Trizol®Reagent (Invitrogen) and was resuspended in DEPC-treated water. The quality of the RNA was assessed by electrophoresis on a 1% agarose gel based on the integrity of the 28S and 18S RNA bands, its concentration was estimated by measuring the absorbance at 260 nm and its purity was varied from the 260/280 nm absorption ratio. Only samples with absorption ratios in the range of 1.8 to 2.0 were used in reverse transcription reactions, in which one microgram of total RNA was incubated with DNaseI (Invitrogen) and then reverse-transcribed into cDNA using oligo-d(T)18 primers and the superscript III reverse transcriptase kit (Invitrogen) according to the manufacturer's instructions.

**6. Subcloning, production of adenoviruses and infection of CMs by adenoviruses**

The sequences of AT_1_R and AT_2_R were cloned by PCR reaction from first strand cDNA using mouse liver total RNA. PCR primers were designed based on a published nucleotide sequence of mouse AT_1_R (GenBank accession no. NM_177322.3) and AT_2_R (GenBank accession no. NM_007429.5). For PCR of AT_1_R, forward primer was 5'-CCGCTCGAGGCC ACCAGCAAAATGGCCCTTAACTCTTC-3', while reverse primer was 5'-CCCAAG CTTCTCCACCTCAGAACAAGACGCAGG-3'. For PCR of AT_2_R, forward primer was 5'- CCGCTCGAGGCCACCCAGTTGCTGCAGTTCAATATGAAG-3', while reverse primer was 5'-CCCAAGCTTCACAAAGGTGTCCATTTCTCTAAG-3'. The PCR products of AT_1_R and AT_2_R were respectively inserted into pEYFP-N1 (kindly provided by Professor Liwen Jiang's laboratory) to obtain AT_1_R-YFP fusion construct and AT_2_R-YFP fusion construct. For construction of pShuttle-CMV-AT_1_R-YFP and pShuttle-CMV-AT_2_R-YFP, pShuttle-CMV plasmid [3], which was a gift from Professor Bert Vogelstein (Addgene plasmid # 16403), was used. pShuttle-CMV plasmid was double-digested with the enzymes Bg III and Not I and the AT_1_R-YFP fusion construct and AT_2_R-YFP fusion construct were double-digested with the same two enzymes. Digested pShuttle-CMV backbone was then ligated with the digested fusion construct. The resultant pShuttle-CMV-AT_1_R-YFP and pShuttle-CMV-AT_2_R-YFP were then linearized by digestion with PmeI. The linear plasmids were transformed into BJ5183 competent cells with pAdEasy-1 for homologous recombination. After screening on LB solid culture plates with 100 μg/mL kanamycin and identification, pAdTrack-CMV-AT_1_R-YFP and pAdTrack-CMV-AT_2_R-YFP were ready and stored at -20 °C.

HEK293AD cells were grown up to 70% confluence and were then respectively transfected with adenovirus vectors pAdTrack-CMV-AT_1_R-YFP and pAdTrack-CMV-AT_2_R-YFP, which were linearized with PacI. After 24 hrs, cells were checked for yellow fluorescent protein (YFP) expression, and about 1 week after the transfection, adenoviruses Ad-AT_1_R and Ad-AT_2_R were collected. HEK293AD cells were scraped into vials for freeze-and-thaw cycles for 3 times, and the vials were then centrifuged for 5 mins at 12,000 rpm. Supernatant containing the adenoviruses Ad-AT_1_R and Ad-AT_2_R were collected for subsequent infection. After repeating this infection and collection procedure 3 times to amplify the primary stock, Ad-AT_1_R and Ad-AT_1_R were used for infecting target cells.

Single CMs were infected with adenovirus expressing YFP, AT_1_R, AT_2_R for 12 hrs at 100 MOI. Thereafter, the medium was changed back to normal differentiation medium. Infection efficiency was determined 36 - 48 hrs after infection by YFP fluorescence intensity and was typically assessed to be 90 - 95%.

**7. Immunocytochemistry**

Cells on glass coverslip were fixed in 4% paraformaldehyde (Sigma) for 15 mins. Cells were rinsed in PBS twice for 5 mins, and subsequently incubated in 0.1% Triton X-100 (Sigma) for 15 mins. Cells were washed with PBS twice, and incubated in blocking solution containing 0.5 % milk and 5 % normal goat serum (NGS) for 1 hr. Cells were incubated with primary antibodies [cardiac troponin T (cTnT) 1:500 (Abcam, Cambridge, UK); TRPC3 1:100 (Abcam); AT_1_R 1:100 (Abcam); AT_1_R 1:100 (Alomone, Jerusalem, Israel); AT_2_R 1:100 (Abcam); AT_2_R 1:100 (Santa Cruz, Dallas, TX, USA); Ang II 1:250 (Peninsula Laboratories, Merseyside, UK); sarcoplasmic/endoplasmic reticulum Ca^2+^-ATPase isoform 2 (SERCA2) 1:200 (Abcam)] at 4 ^o^C overnight. After being washed four times for 5 mins with 0.1 % tween-20, the cells were incubated with 1% NGS solution with Dylight 488-conjugated goat anti-mouse IgG (1:100; Jackson ImmunoResearch, West Grove, PA, USA) or Dylight 594-conjugated goat anti-rabbit IgG (1:100; Jackson ImmunoResearch) for 1 hr at room temperature. Cells were washed four times with 0.1 % tween-20. For staining with AT_2_R-ATTO-488 antibody (Alomone), cells on glass coverslip were fixed in 4 % paraformaldehyde for 10 mins. Cells were rinsed in PBS twice for 5 mins, and subsequently incubated in 0.1 % Triton X-100 for 15 mins. Cells were washed with PBS twice, and incubated in blocking solution containing 1 % bovine serum albumin (Sigma) for 1 hr. Antibodies were applied at 1:50. Images were acquired using Olympus FluoView FV1000 confocal laser scanning microscope with 60X objective and analyzed using the FV1000 software.

## 8. Total protein extraction

Cells were lysed in ice-cold RIPA buffer with 1 % Nonidet P-40, 0.1 % SDS, 0.5 % sodium deoxycholate in PBS (Invitrogen), freshly supplemented with protease inhibitor cocktail [leupeptin (1 μg/mL), aprotinin (5 μg/mL), PMSF (0.59 mM), sodium orthovanadate (1 mM), EGTA (1 mM), EDTA (1 mM)] and phosphatase inhibitor cocktail [NaF (1.6 mM), β-glycerolphosphate (4 mg/mL) and HEPES (0.4 mM, pH 7.3)] and placed on ice for 15 mins. Cells were centrifuged at 16,000 g at 4 ^o^C for 20 mins. Supernatant that contained protein was stored at -80 ^o^C. Protein concentration was determined by Bradford assay with BSA as the standards.

**9. Western blot**

50 μg total proteins mixed 1:1 (v:v) with 2x sample buffer [139 mM Tris (pH 6.8), 4.4 % SDS, 22.2 % glycerol, 0.07 % bromophenol blue (Bio-Rad, Hercules, CA, USA)] supplemented with 10 % β-mercaptoethanol (v/v) (Sigma), were separated in 7.0 % SDS polyacrylamide gel and were transferred to 0.45 μm PVDF membranes (Millipore). Membrane was blocked for 1 hr at room temperature with 5 % (w:v) non-fat dry milk (Bio-Rad) in TBST (50 mM Tris, 150 mM NaCl, 0.05 % Tween 20), and incubated with primary antibodies at 4 °C overnight. Anti-AT_2_R antibody (Alomone) was used at 1:500. To determine the specific of anti-AT_2_R antibody, peptide preincubation experiment was performed. Control peptide antigen and anti-AT_2_R were mixed at a ratio of 5:1 in PBS with shaking for 2 hrs at 37 °C. The mixture was centrifuged at 10,000 g for 15 mins prior to being used on the membrane. After washing with TBST, membranes were then incubated with horseradish peroxidase-conjugated goat anti-rabbit secondary antibody (Dako, Santa Clara, CA, USA) at 1:4000 for 1 hr at room temperature. After the membrane was washed with TBST, protein signals were developed by using a Western Lightening Plus-ECL Kit (Perkin Elmer, Waltham, MA, USA).

**10. Quantification of iAng II levels in NRVMs using UHPLC-ESI-MS/MS**

*10.1 Total protein extraction*

NRVMs (2x10^6^ cells), from 10 neonatal rats, were lysed in ice-cold RIPA buffer, freshly supplemented with protease inhibitor cocktail and placed on ice for 15 mins. NRVMs were centrifuged at 16,000 g at 4 ^o^C for 20 mins. Supernatant (300 μL) that contained protein was stored at -80 ^o^C, as the next procedure sample. Protein concentration was determined by Bradford assay with BSA as the standards.

*10.2 Purification and concentration of iAng II*

Sample (300 μL) was added into 5 mL of 1 % heptafluorobutyric acid (HFBA) (Thermo Fisher Scientific, Waltham, MA, USA) and centrifuged at 3000×g for 15 min at 4 °C. Subsequently, 4 mL of supernatant was taken into glass tube and evaporated to 1 mL. In addition, the concentrated sample (1 mL) was mixed with 9 mL of 0.1% HFBA for purification and concentration of iAng II by C18 Sep-Pak Vac 3cc cartridges (200 mg) (Waters, Germany). At first, the Sep-Pak cartridges were preconditioned with 5 mL of 80% (v/v) MeOH in H_2_O containing 0.1% (v/v) HFBA (80 mL of MeOH, 20 mL of H_2_O, and 100 μL of HFBA) followed by 10 mL of 0.1 % HFBA (to avoid drying out of the column). Next, the sample was loaded. The cartridge was washed with 10 mL 0.1 % HFBA followed by 5 mL LC-MS grade water. The samples were eluted from the SPE cartridge with 3 mL MeOH. The eluate was evaporated to dryness, reconstituted with 300 μL PBS for next immunoaffinity purification.

*10.3 Immunoaffinity purification of iAng II*

Anti-Ang II antibody (80 μg, 200 μL) was diluted in 1 mL of coupling buffer (0.5 M NaCl, 0.1 M NaHCO3, pH 8.3) prior to immobilization. 200 mg of CNBr-activated Sepharose 4B (GE Healthcare, Uppsala, Sweden) was swelled in 1 mL of HCl (1 mM) to remove the protecting groups. The diluted antibody was immediately transferred to the washed resin. The immobilization was carried out for 1 h at room temperature on an overhead rotor. After immobilization, the unbound substrate was washed out three times using 6 mL of coupling buffer. The supernatant was removed and the unbound reactive groups of the sepharose resin were blocked using blocking buffer (0.1 M Tris-HCl, pH 8.0) for 2 h at room temperature. Next, the resin was alternatively washed with 3 cycles of 5 mL of both wash buffer 1 (0.1 M NaCH_3_COO, 0.5 M NaCl, pH 4.0,) and wash buffer 2 (0.5 M NaCl, 0.1 M Tris-HCl, pH 8). The immobilized anti-Ang II antibodies were stored in PBS containing 0.02% NaN_3_ at 4°C until use. Prior to use, the immobilized anti-Ang II antibodies were washed five times using 1 mL PBS. For further immunoaffinity purification of iAng II, the extracted samples were incubated for 1 hr at 4 °C with 30 μL of immobilized anti-Ang II antibodies. Next, the beads were washed two times using 500 μL PBS. In order to remove all salt, the resin was washed two times using 500 μL LC-MS grade water. Ang II was eluted three times using 40 μL of 0.1% formic acid directly into the glass vial and evaporated to dryness. The dried samples were reconstituted in 40 μL 0.1% formic acid and analyzed by UHPLC-ESI-MS/MS.

*10.4 LC-MS/MS analysis*

All LC-MS/MS analysis were performed on a Ultimate 3000 UHPLC system coupled to a Thermo TSQ mass spectrometer (Thermo Fisher Scientific). The column oven was maintained at 30 ^o^C. The flow rate was set to 0.3 mL/min. The mobile phases were ultrapure water with 0.1% formic acid (mobile phase A) and acetonitrile (mobile phase B). The sample injection volume was 10 μL in the positive ion mode. The elution gradient is shown in Supplementary Table 1. The capillary temperature was set at 300 ^o^C; spray voltage was 3.5 kV; heater temperature was set at 350 ^o^C; sheath gas flow rate was 30 arb; aux gas flow rate was 10 arb; the mass scan range was m/z 100-1100. The MS/MS analysis was acquired in targeted MS/MS mode with 2 collision energies of 16.8 eV and 18.3 eV, respectively. The accumulation time was 100 ms. The double charged parent ions of the native Ang II was m/z 523.8±0.1 (Supplementary Fig. 1A). The structure of Ang II was confirmed by two kinds of MRM transitions (Supplementary Fig. 1B,C). The both fragment ion m/z 784.4±0.1 and m/z 263.1±0.1 were optimized for the detection (Supplementary Fig. 1C). The latter fragment ion was the dominant ion with the highest intensity and therefore was chosen to be used for quantification. The isolation and the detection of the both ions were performed within the same scan. For the MS/MS mode, helium was uses as the collision gas. The quantification the extracted ions chromatograms for the ion was 263.1±0.1 m/z and the qualitative ion was 784.4±0.1 m/z. The best collision energy was between 10 ~ 30 V. The MRM transitions and the best collision energy were shown in Supplementary Table 2.

*10.5 Method application*

The new method was applied for quantification of Ang II level in heart. The identity of the native Ang II was confirmed using both the retention time and the fragment ion. Extracted ion chromatograms of the sample were generated. The quantitative levels of the native Ang II were also calculated using concentration and peak-area ratio. (see "Results" section).

**11. Calcium transient imaging**

Single spontaneously beating mESC-CMs were obtained as described above. Tyrode’s solution (1 mM MgCl_2_, 1.8 mM CaCl_2_, 5.4 mM KCl, 10 mM glucose, 10 mM HEPES and 140 mM NaCl, pH adjusted to 7.2 by NaOH) was used to wash the cells before imaging. After incubation with 5 µM Fluo-4 (Invitrogen) and 0.02 % pluronic acid F-127 (Sigma) in dark for 12 minutes at 37 ^o^C, mESC-CMs were gently washed with Tyrode’s solution again. Images were obtained using Olympus FluoView FV1000 confocal laser scanning microscope (Olympus, Japan) equipped with an argon laser of 488 nm at a frequency of 59 Hz. Raw traces of CaTs were shown as fluorescence intensity. Data analysis was performed with FV1000 (Olympus) and Origin 7.0 software (OriginLab, Northampton, MA, USA) [4]. To quantify the rate of recovery of intracellular calcium and therefore the decay time, the decay portion of the CaTs (from 30% to 100% of the decline phase) was measured by the time constant (τ) of a single exponential fit [5]. The percentage change in different parameters of the CaTs upon different treatments was calculated by normalizing the values to that of the basal level before the application of solvent or drugs.

**12. Drugs**

Ang II, lorsartan and chymostatin were purchased from Sigma. PD123319 and Pyr3 were purchased from Tocris Cookson (Bristol, UK). Benazeprilat was purchased from Santa Cruz Biotechnology (Dallas, TX, USA). Compound 21 (C21) was a kind gift from Vicore Pharma (Göteborg, Sweden). Water was used as the solvent for Ang II, losartan, PD123319, and C21 while DMSO was used as the solvent for benazeprilat and chymostatin.

**REFERENCES**

1 Wobus, A.M.; Guan, K.; Yang, H.T.; Boheler, K.R.. Embryonic stem cells as a model to study cardiac, skeletal muscle, and vascular smooth muscle cell differentiation. In: *Turksen K. Methods in Molecular Biology, vol 185: Embryonic Stem Cells: Methods and Protocols. New York: Springer* **2002**, 127-156.

2 Wollert, K.C.; Taga, T.; Saito, M.; Narazaki, M.; Kishimoto, T.; Glembotski, C.C.; Vernallis, A.B.; Heath, J.K.; Pennica, D.; Wood, W.I.; Chien, K.R*.* Cardiotrophin-1 activates a distinct form of cardiac muscle cell hypertrophy. Assembly of sarcomeric units in series VIA gp130/leukemia inhibitory factor receptor-dependent pathways. *J Biol Chem* **1996**, 271, 9535-9545.

3 He, T.C.; Zhou, S.; da Costa, L.T.; Yu, J.; Kinzler, K.W.; Vogelstein, B*.* A simplified system for generating recombinant adenoviruses. *Proc Natl Acad Sci USA* **1998**, 95, 2509-2514.

4 Wong, C.K.; So, W.Y.; Law, S.K.; Leung, F.P.; Yau, K.L.; Yao, X.; Huang, Y.; Li, X.; Tsang, S.Y. Estrogen controls embryonic stem cell proliferation via store-operated calcium entry and the nuclear factor of activated T-cells (NFAT). *J Cell Physiol* **2012**, 227, 2519-2530.

5 Laurita, K.R.; Katra, R.; Wible, B.; Wan, X.; Koo, M.H. Transmural heterogeneity of calcium handling in canine. *Circ Res* **2003**, 92, 668-675.

**SUPPLEMENTARY FIGURE LEGENDS**

**Figure S1**

Detection of Ang II by UHPLC-ESI-MS/MS. (A) The positive full scan spectrum of native Ang II by UHPLC-ESI-MS/MS. The one, double and three charge parent ions were 1046.4±0.1 m/z, 523.8±0.1 m/z and 349.6±0.1 m/z respectively. (B) The extracted ion chromatograms of the two kinds of MRM. The upper one was the 523.8±0.1 m/z 🡪263.1±0.1 m/z; the lower one was the 523.8±0.1 m/z 🡪784.4±0.1 m/z. (C) The positive MS/MS spectrum of the parent ion 523.8±0.1 m/z.

**Figure S2**

AT_1_R and AT_2_R have different subcellular localizations in NRVMs.(A) NRVMs were transduced with vector carrying AT_1_R-YFP and were stained with DAPI (blue). AT_1_R-YFP signals were detected on the plasma membrane. (B) NRVMs were stained with DAPI (blue), anti-cTnT (red), and anti-AT_2_R (green). (C) Representative Western blot analysis of AT_2_R in NRVMs. Lane 1 represents proteins from NRVMs probed with anti-AT_2_R, while lane 2 represents the same protein lysates but probed with anti-AT_2_R that was preincubated with the control peptide antigen.

**Figure S3**

AT_1_R blocker attenuated Ang II-induced changes in CaTs. (A) Raw traces of spontaneous CaTs at basal level and (upper panel) upon treatment with 0.1 µM Ang II, or (lower panel) upon treatment with 100 μM losartan followed by the subsequent application of 0.1 µM Ang II in mESC-CMs. Summarized data on the (B) amplitude, (C) time-to-peak, (D) frequency and (E) decay time of CaTs upon treatment with 0.1 µM Ang II alone or upon treatment with 100 μM losartan followed by 0.1 µM Ang II. Losartan attenuated Ang II-induced changes in the parameters of CaTs. Values are mean ± SEM of 4-6 independent experiments. ** *P* < 0.01, *** *P* < 0.001.

**Figure S4**

Solvent control had no effect on APs of mESC-CMs by dual patch clamp. (A) Dual current patch clamp of APs of mESC-CMs without intracellular drug delivery (i.e. before membrane breakage by the 'drug electrode'). Upper panel represents recording in 'drug electrode' while lower panel represents recording in 'recording electrode'. (B) Dual current patch clamp of APs of mESC-CMs with intracellular delivery of solvent (i.e. after membrane breakage by the ‘drug electrode’). Upper panel represents recording in 'drug electrode' while lower panel represents recording in 'recording electrode'. (C) Traces labeled as pink in (A) (represents APs before solvent delivery) and as blue in (B) (represents APs after intracellular solvent delivery) are overlaid for comparison. Summarized data on the (D) AP rate, (E) DD slope, (F) APD 50, (G) MDP of APs upon treatment with solvent. Values are mean ± SEM of 6-10 independent experiments.

**Figure S5**

Intracellular delivery of AT_1_R blocker losartan exerted no effect on the APs of mESC-CMs. (A) Dual current patch clamp of APs of mESC-CMs without intracellular drug delivery (i.e. before membrane breakage by the 'drug electrode'). Upper panel represents recording in 'drug electrode' while lower panel represents recording in 'recording electrode'. (B) Dual current patch clamp of APs of mESC-CMs with intracellular delivery of losartan (i.e. after membrane breakage by the ‘drug electrode’). Upper panel represents recording in 'drug electrode' while lower panel represents recording in 'recording electrode'. (C) Traces labeled as pink in (A) (represents APs before intracellular losartan delivery) and as blue in (B) (represents APs after intracellular losartan delivery) are overlaid for comparison. Summarized data on the (D) AP rate, (E) DD slope, (F) APD 50, (G) MDP of APs upon treatment with intracellular losartan. Values are mean ± SEM of 6-10 independent experiments.

**Figure S6**

Intracellular delivery of AT_2_R blocker PD123319 (10 µM) exerted no effect on the APs of mESC-CMs. (A) Dual current patch clamp of APs of mESC-CMs without intracellular drug delivery (i.e. before membrane breakage by the 'drug electrode'). Upper panel represents recording in 'drug electrode' while lower panel represents recording in 'recording electrode'. (B) Dual current patch clamp of APs of mESC-CMs with intracellular delivery of PD123319 (i.e. after membrane breakage by the ‘drug electrode’). Upper panel represents recording in 'drug electrode' while lower panel represents recording in 'recording electrode'. (C) Traces labeled as pink in (A) (represents APs before intracellular PD123319 delivery) and as blue in (B) (represents APs after intracellular PD123319) are overlaid for comparison. Summarized data on the (D) AP rate, (E) DD slope, (F) APD 50, (G) MDP of APs upon treatment with intracellular PD123319. Values are mean ± SEM of 6-10 independent experiments.

**Figure S7**

iAng II decreased the APs of NRVMs in a persistent manner. (A) Dual current patch clamp of APs of NRVMs without intracellular drug delivery (i.e. before membrane breakage by the 'drug electrode'). Upper panel represents recording in 'drug electrode' while lower panel represents recording in 'recording electrode'. (B) Dual current patch clamp of APs of NRVMs with intracellular delivery of Ang II (i.e. after membrane breakage by the ‘drug electrode’). Upper panel represents recording in 'drug electrode' while lower panel represents recording in 'recording electrode'. (C) Traces labeled as pink in (A) (represents APs before iAng II delivery) and as blue in (B) (represents APs after intracellular iAng II delivery) are overlaid for comparison. Summarized data on the (D) AP rate, (E) DD slope, (F) APD 50, (G) MDP of APs upon treatment with iAng II. Values are mean ± SEM of 6-10 independent experiments. * *P* < 0.05 Vs control group.

**Figure S8**

Intracellular delivery of AT_2_R activator C21 decreased the APs of NRVMs. (A) Dual current patch clamp of APs of NRVMs without intracellular drug delivery (i.e. before membrane breakage by the 'drug electrode'). Upper panel represents recording in 'drug electrode' while lower panel represents recording in 'recording electrode'. (B) Dual current patch clamp of APs of NRVMs with intracellular delivery of C21 (i.e. after membrane breakage by the ‘drug electrode’). Upper panel represents recording in 'drug electrode' while lower panel represents recording in 'recording electrode'. (C) Traces labeled as pink in (A) (represents APs before intracellular C21 delivery) and as blue in (B) (represents APs after intracellular intracellular C21 delivery) are overlaid for comparison. Summarized data on the (D) AP rate, (E) DD slope, (F) APD50, (G) MDP of APs upon treatment with intracellular C21. Values are mean ± SEM of 6-10 independent experiments. * *P* < 0.05 Vs control group.

**Figure S1**


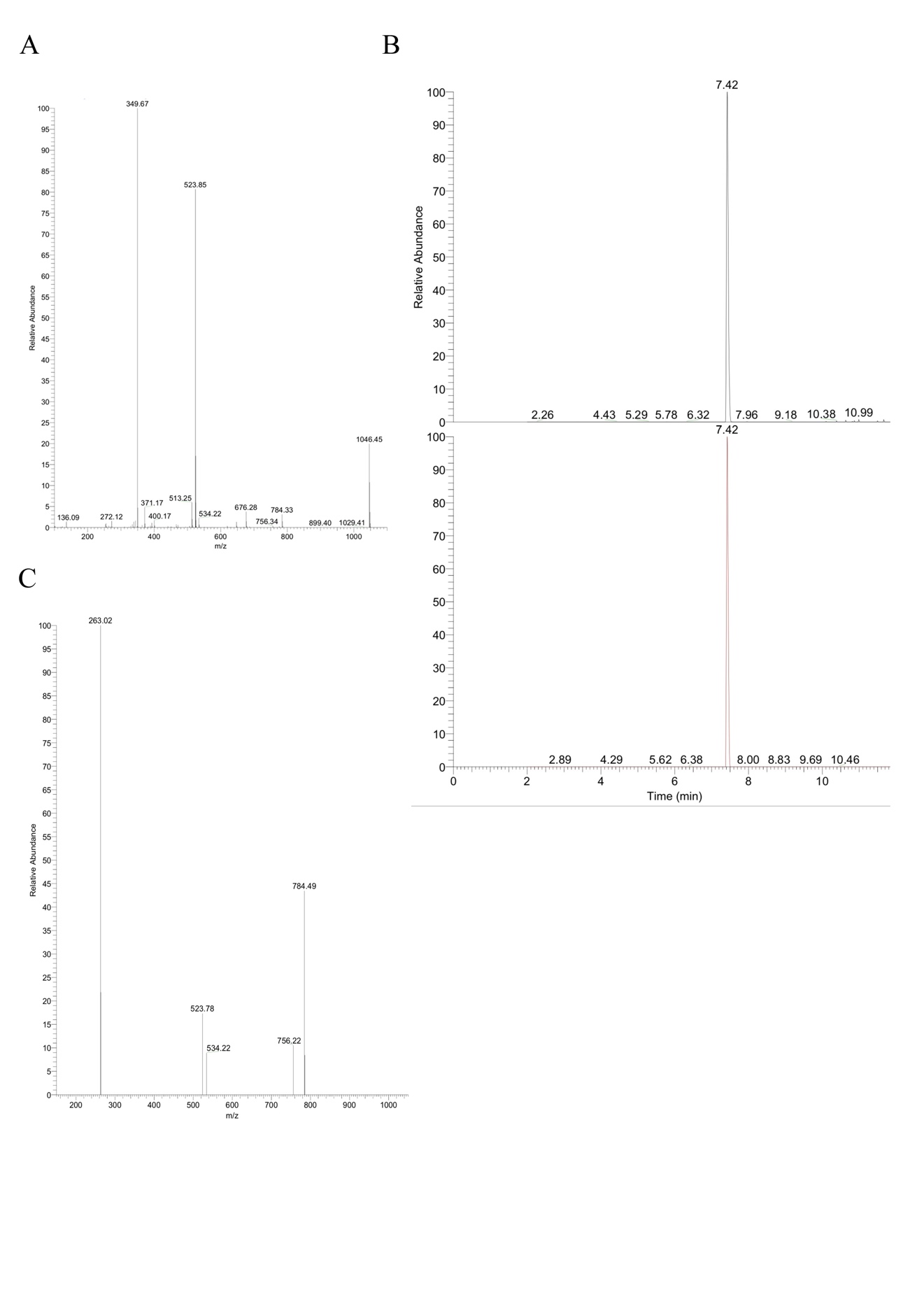


**Figure S2**


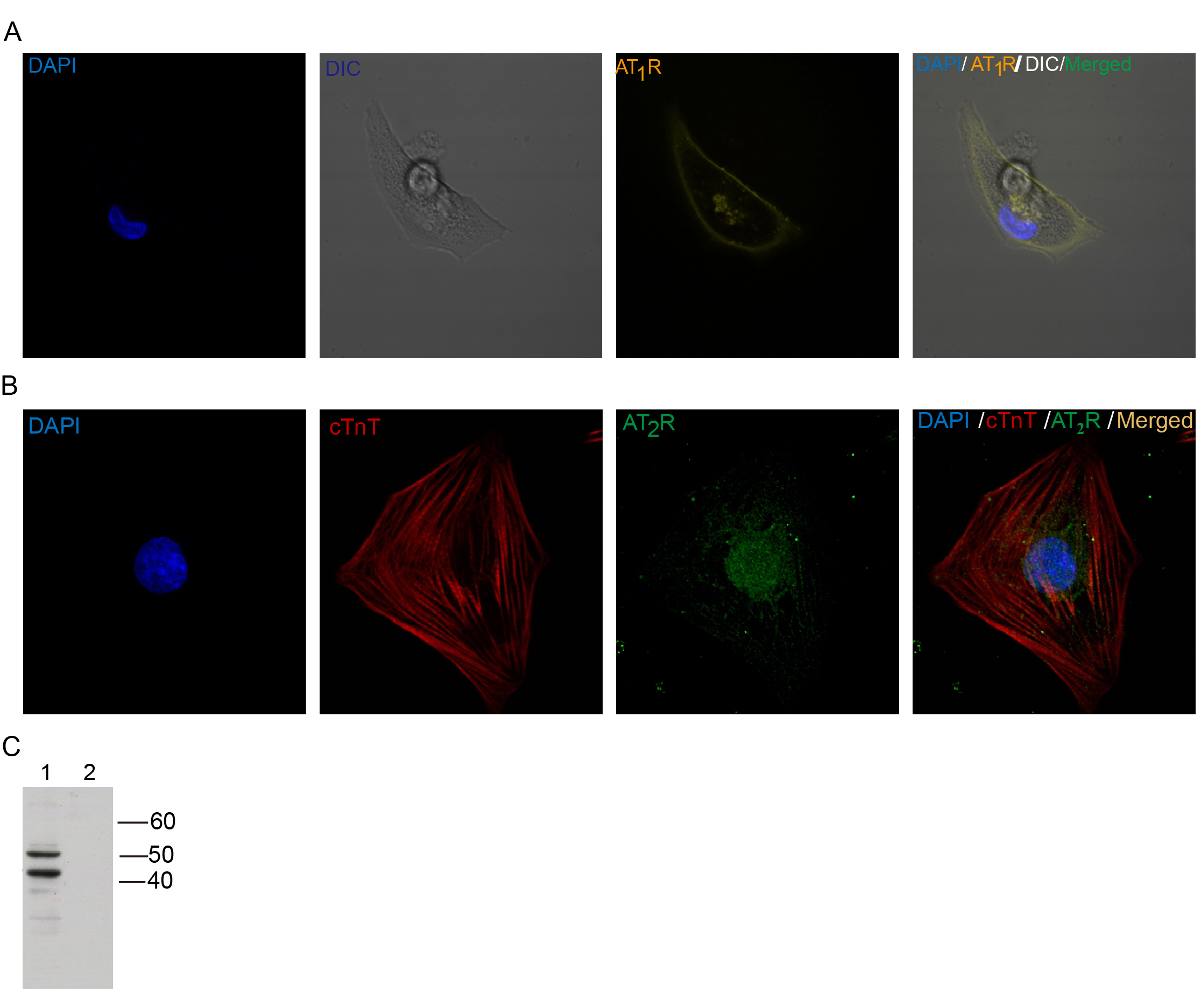


**Figure S3**


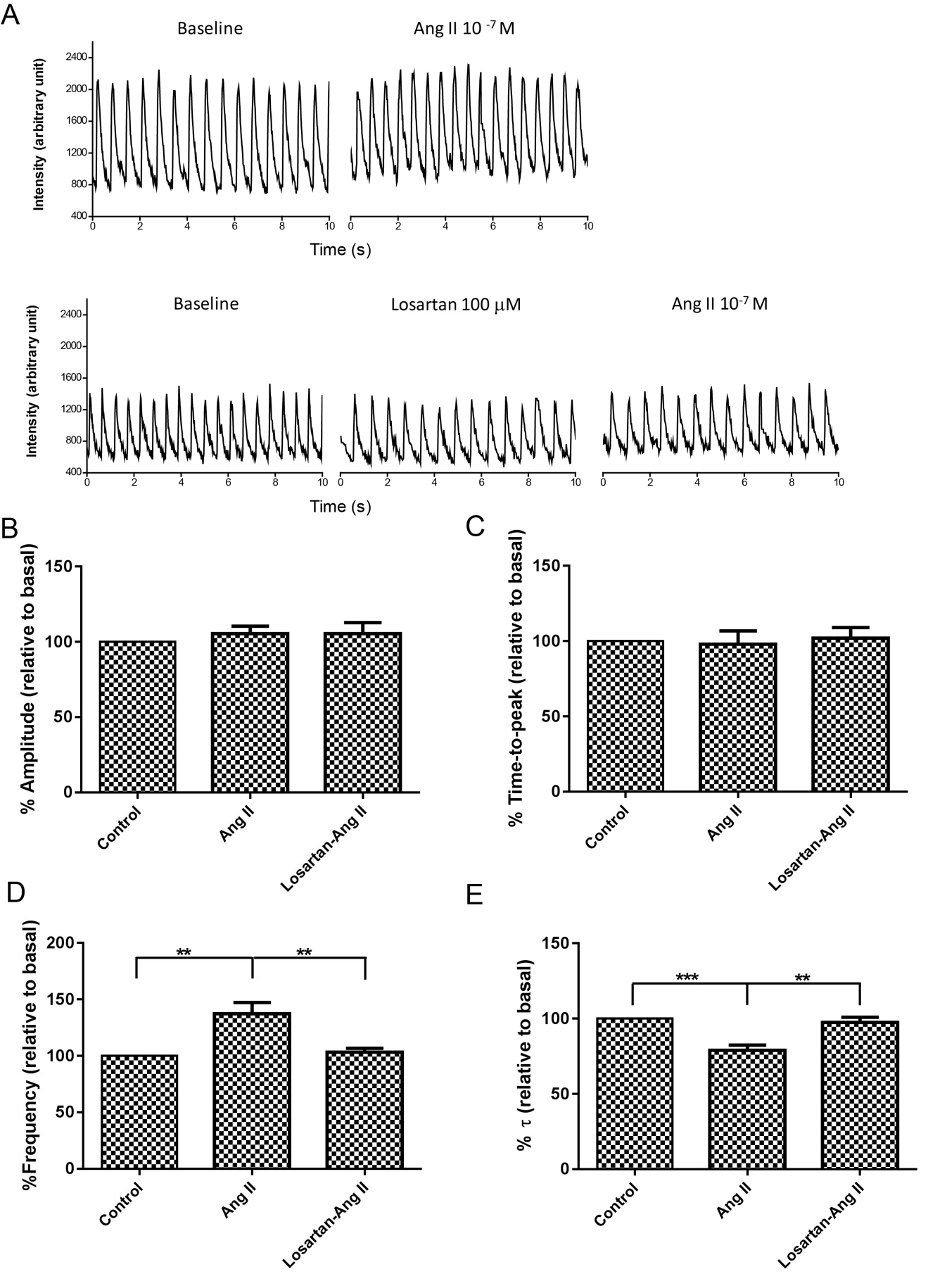


**Figure S4**


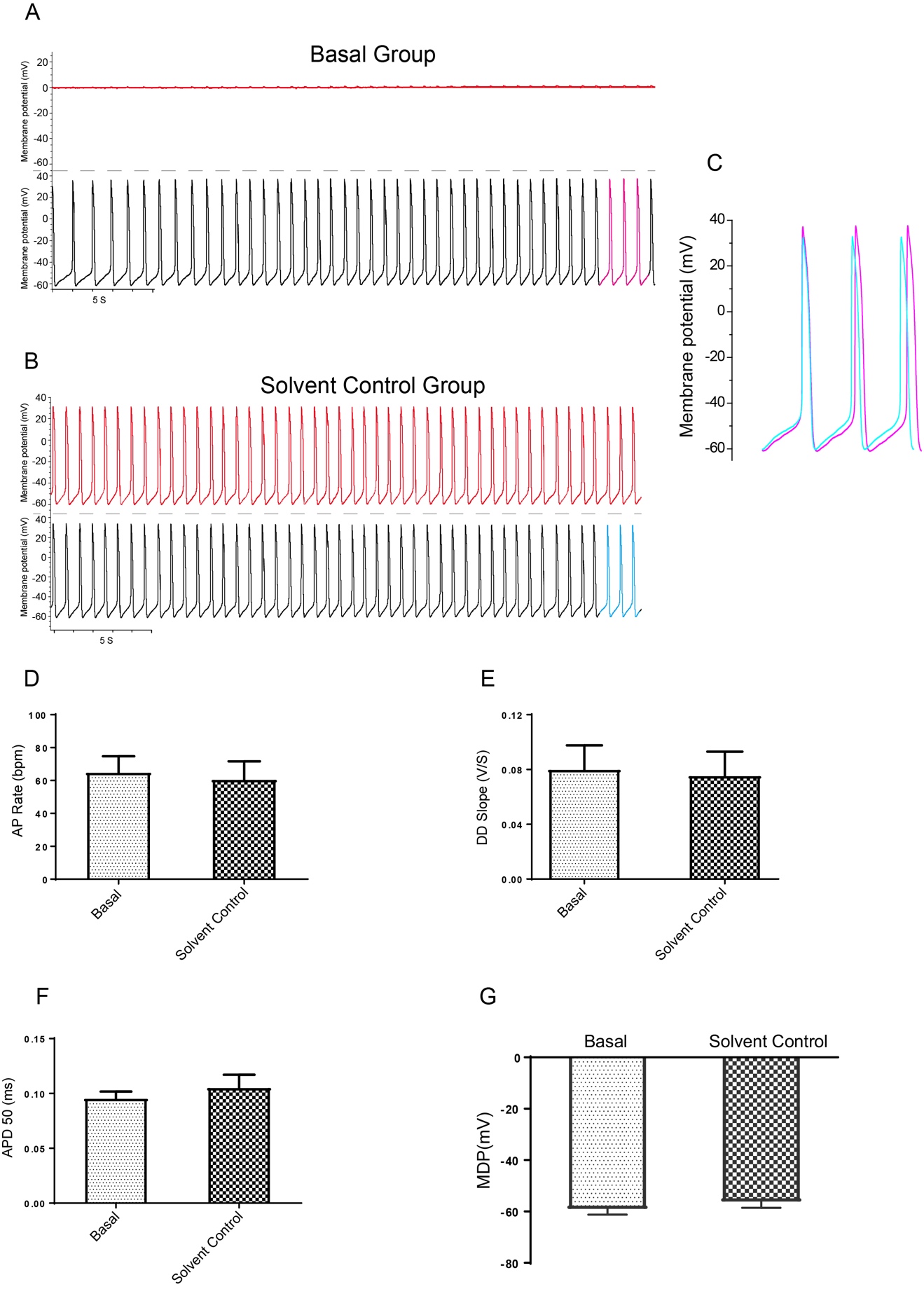


**Figure S5**


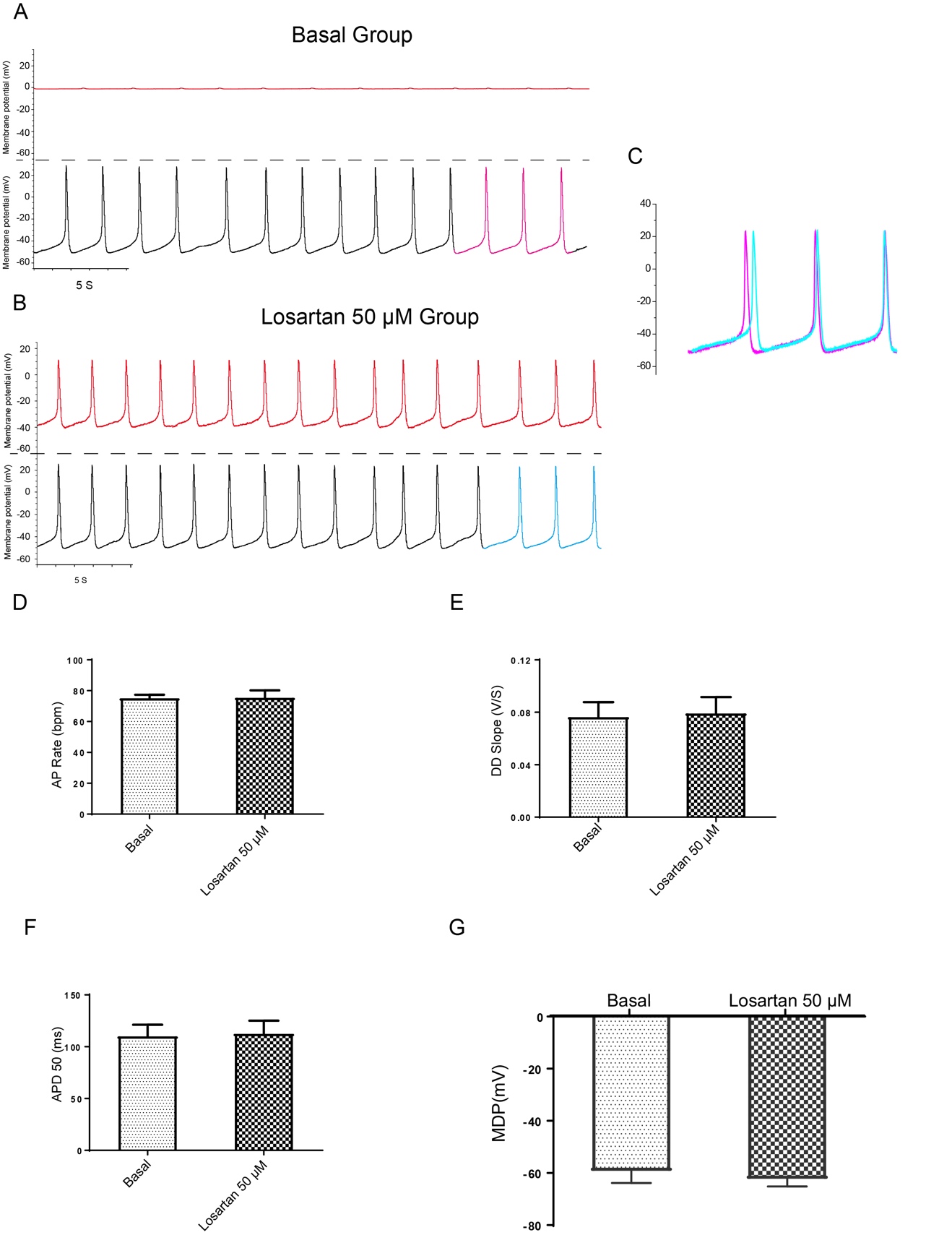


**Figure S6**


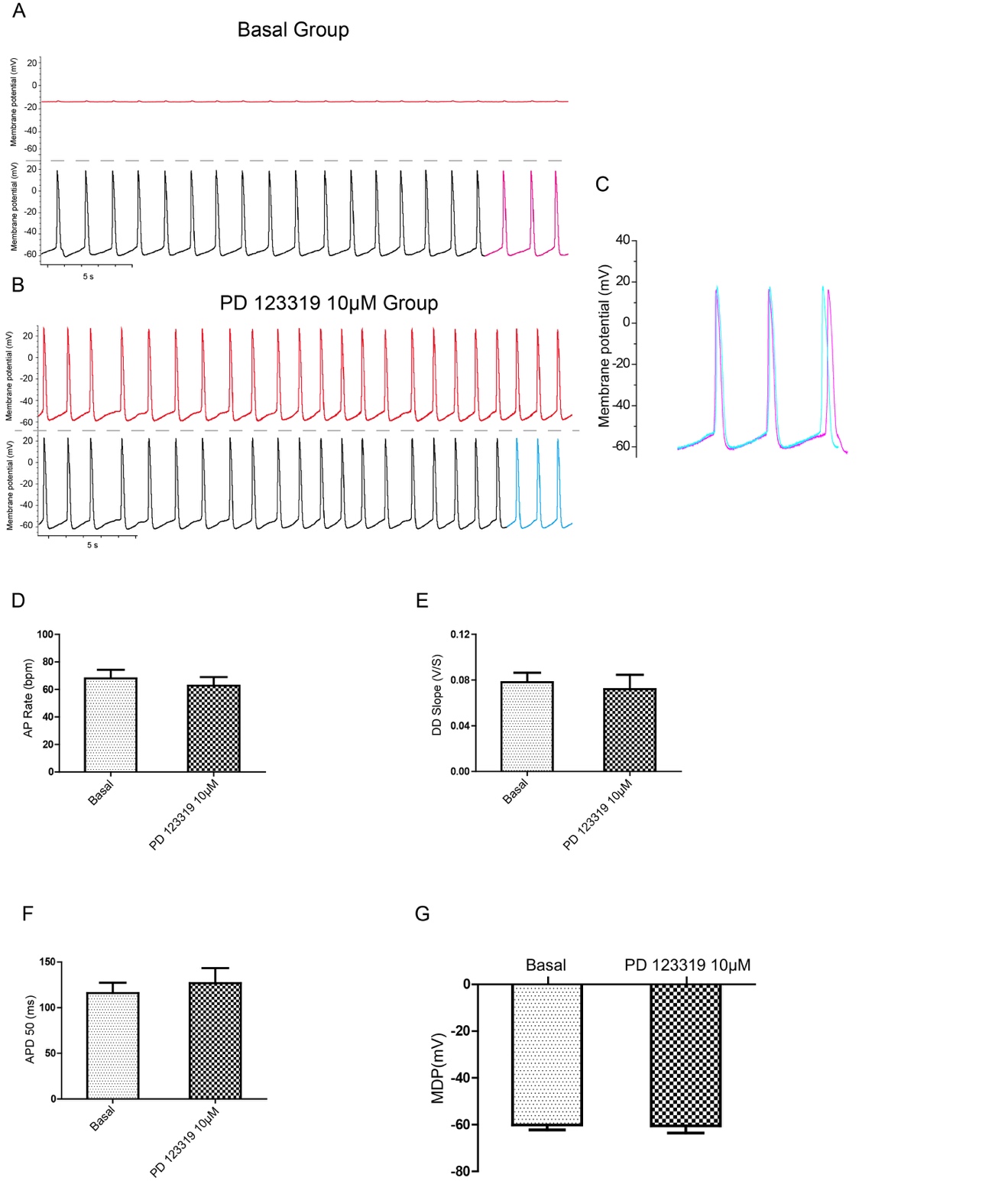


**Figure S7**


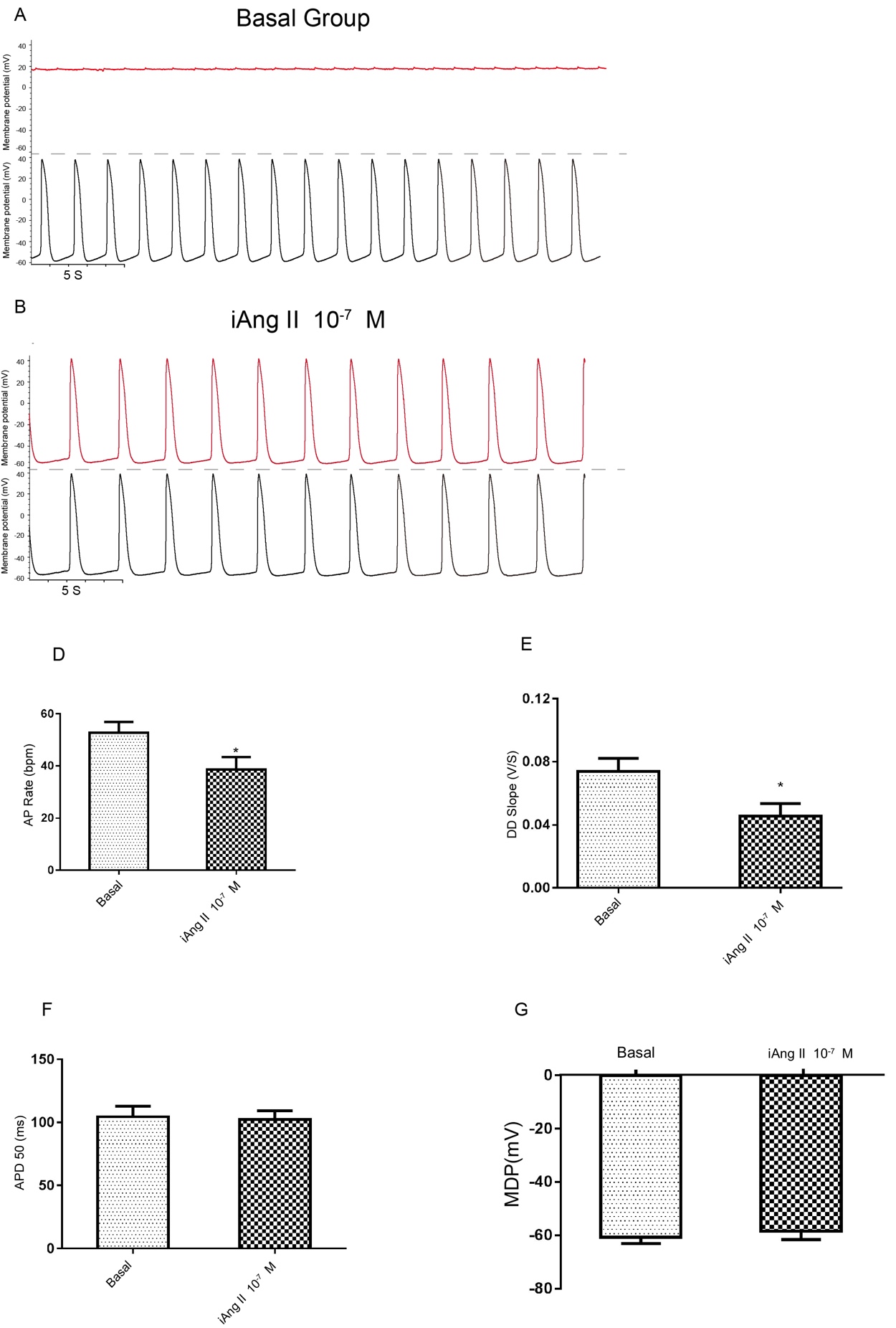


**Figure S8**


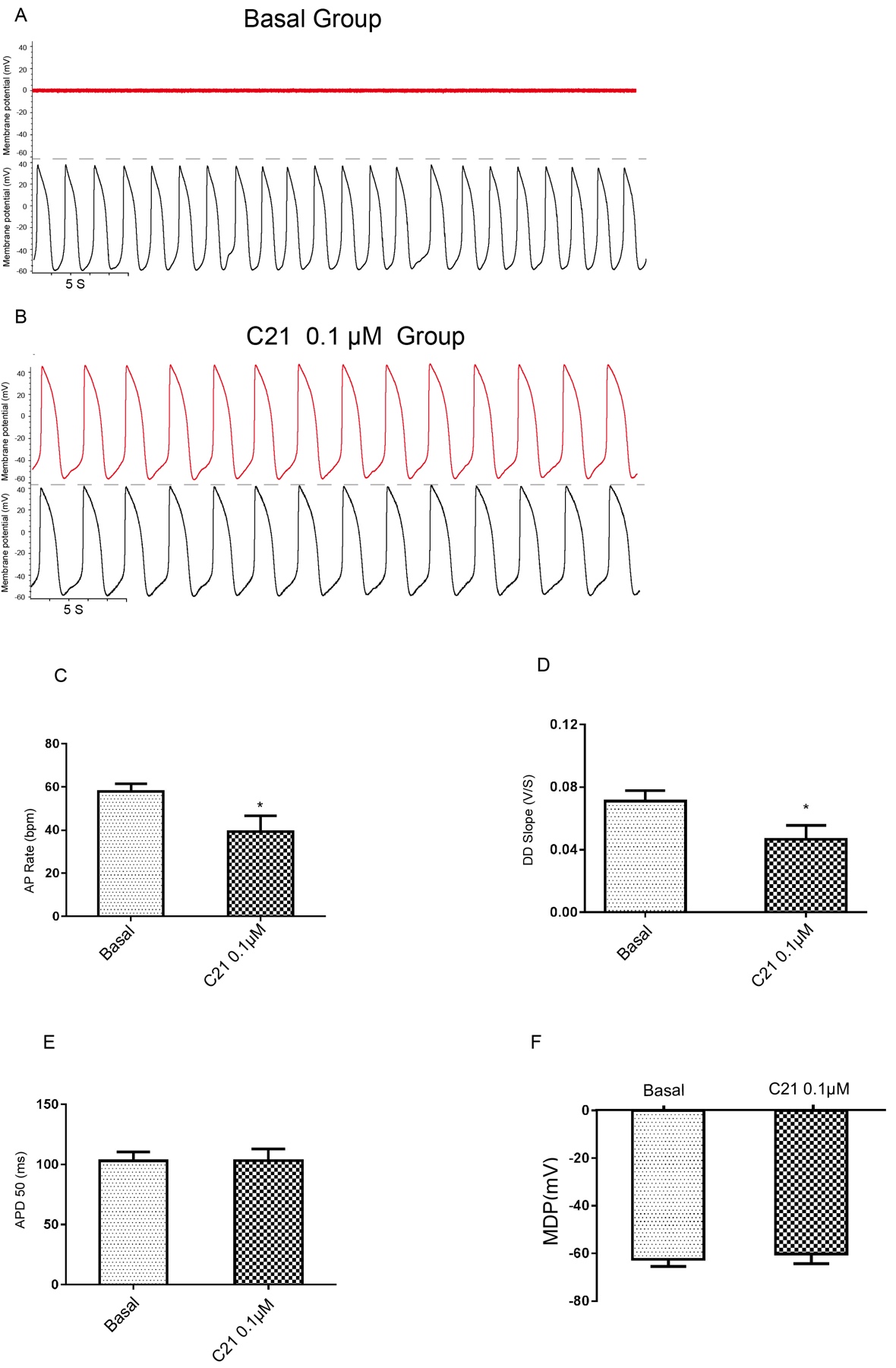


Supplementary Table 1. LC elution gradient in positive mode.

| Positive ion mode | | |
| --- | --- | --- |
| Time (min) | A% | B% |
| 0 | 98 | 2 |
| 5 | 80 | 20 |
| 9 | 0 | 100 |
| 10 | 0 | 100 |
| 10.5 | 98 | 2 |
| 13 | 98 | 2 |

Supplementary Table 2. Compound Optimization Results

| Precursor  m/z | Product  m/z | Collision Energy (V) | RF Lens (V) | Intensity |
| --- | --- | --- | --- | --- |
| 523.861 | 263.111 | 18.343 | 98.225 | 26492.448 |
| 523.861 | 784.333 | 16.876 | 98.225 | 9795.32 |
